# 2-Methyl Nonyl Ketone From *Houttuynia Cordata Thunb* Alleviates LPS-Induced Inflammatory Response and Oxidative Stress in Bovine Mammary Epithelial Cells

**DOI:** 10.3389/fchem.2021.793475

**Published:** 2022-01-31

**Authors:** Nan Wang, Yanbin Zhu, Dandan Li, Wangdui Basang, Yiqiu Huang, Kening Liu, Yuxin Luo, Lu Chen, Chunjin Li, Xu Zhou

**Affiliations:** ^1^ College of Animal Sciences, Jilin University, Changchun, China; ^2^ Tibet Academy of Agricultural and Animal Husbandry Sciences, Institute of Veterinary and Animal Husbandry, Lhasa, China; ^3^ State Key Laboratory of Hulless Barley and Yak Germplasm Resources and Genetic Improvement, Lhasa, China; ^4^ Reproductive Medical Center, Department of Obstetrics and Gynecology, The Second Hospital of Jilin University, Changchun, China

**Keywords:** 2-methyl nonyl ketone, mastitis, imflammation, oxidative stress, LPS

## Abstract

Mastitis is one of the most common diseases in dairy cows, causing huge economic losses to the dairy industry every year. *Houttuynia Cordata Thunb* (***H.cordata***) is a traditional Chinese herbal medicine that is widely used in clinical treatment. However, the therapeutic effect of 2-methyl nonyl ketone (**MNK**), the main volatile oil component in the aqueous vapor extract of *H. cordata*, on mastitis has been less studied. The purpose of this study was to investigate the protective effect and mechanism of MNK against lipopolysaccharide (**LPS**)-induced mastitis *in vitro*. The results showed that MNK pretreatment of the bovine mammary epithelial cell line (**MAC-T**) enhanced cell viability and inhibited LPS-induced reactive oxygen species (**ROS**) production and inflammatory response. MNK reduced the production of pro-inflammatory cytokines such as interleukin (**IL**) and tumor necrosis factor-α (**TNF-α**) by repressing LPS-induced activation of Toll-like receptor 4-nuclear factor-κB (**TLR4-NF-κB**) signaling pathway. In addition, MNK protected cells from inflammatory responses by blocking the downstream signaling of inflammatory factors. MNK also induced *Heme Oxygenase-1* (***HO-1***) production by Nuclear factor erythroid 2-related factor 2 (**Nrf2**) pathway through AKT and extracellular signal-regulated kinase (**ERK**) pathways, thereby reducing LPS-induced oxidative damage for MAC-T cells. In conclusion, MNK played a protective role against LPS-induced cell injury. This provides a theoretical basis for the research and development of MNK as a novel therapeutic agent for mastitis.

## Introduction

Mastitis is a common infectious disease in dairy cows and is highly influential and easily recurring (Klaas and Zadoks, 2018). Mastitis leads to reduced milk production, lower milk quality, shorter productive life and higher culling rates in dairy cows, causing huge economic losses to dairy farming worldwide (Aghamohammadi et al., 2018; Hadrich et al., 2018; Puerto et al., 2021). Mastitis is an inflammation of the breast caused by pathogenic microorganisms invading the mammary gland and release large amounts of toxins (Klaas and Zadoks, 2018). LPS is a major component of Gram-negative bacteria and can cause severe mammary immune responses. Therefore, LPS is commonly used to construct models of inflammatory response (Guo et al., 2019). The traditional therapy of treating mastitis is antibiotics, but this is prone to cause drug residues and drug resistance. The development of alternative drugs for the prevention and treatment of mastitis is necessary.

**FIGURE 1:**
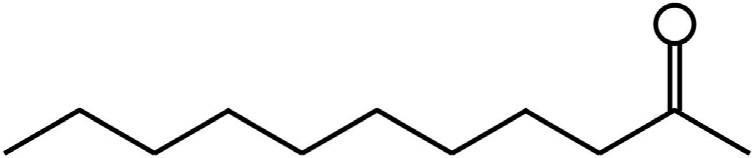
Chemical structure of MNK.

*H.cordata* has various functions such as anti-inflammatory, antibacterial, antiviral, and immune enhancing (Hemalatha et al., 2014). It is rich in volatile oil, alkaloids, flavonoids and other bioactive substances (Li et al., 2017; Chou et al., 2009). Among the components, the volatile oil of *H. cordata* has the highest medicinal value. The drugs prepared from the volatile oil of *H. cordata* has been used in Asia for the treatment of inflammation-related diseases such as diarrhea, conjunctivitis, and respiratory tract infections (Subhawa et al., 2021). MNK (Figure 1) is soluble in ethanol and oil, but not in water. And it is the most abundant and stable component in the volatile oil of *H. cordata*. MNK has been demonstrated to have anti-inflammatory and antioxidant effects (Wu et al., 2021). However, its anti-inflammatory and antioxidant effects in LPS-induced mammary epithelial cells of cows have not been investigated. In this study, MAC-T cells were selected to simulate the inflammation model of mastitis in cows treated with LPS to investigate the protective effect and mechanism of MNK on LPS-induced MAC-T cell injury in cows. The in-depth study of the efficacy of MNK is beneficial to the quality evaluation and control of *H. cordata* and its preparations, as well as provides a basis for natural or synthetic novel anti-inflammatory drugs.

## Materials and Methods

### Cell Culture and Treatment

The MAC-T was cultured in DMEM/F12 1:1 (Hyclone, South Logan, UT, United States) medium supplemented with 10% fetal bovine serum (Gibco, Grand Island, NY, United States) at 37°Cwith 5% CO_2_. After cells were adhered and full grown to about 90% of the cell culture dish (Corning, NY, United States), the cells were digested with 0.25% trypsin (Gibco, CA, United States), collected cells by centrifugation. And then cells were inoculated into different culture dishes and plates (Corning, NY, United States) at the corresponding density for the different assays.

A stock solution of 1 mg/ml LPS (*Escherichia coli* 0111:B4) was stored at 2–8°C (Sigma-Aldrich, St. Louis, MO, United States). LPS was dissolved in phosphate buffer (Gibco, Grand Island, United States). MNK (Yuanye biomart, Shanghai, China) was dissolved in dimethyl sulfoxide (Sigma, St. Louis, MO, United States). A new stock solution of 50 mM MNK was stored at 2–8°C. The cells were pre-treated with MNK (at the concentrations of 0, 5, 10, and 20 µM) for 4 h and then incubated with LPS for 24 h. Cells without any treatment were served as a blank control.

### Cell Viability Assay

The cell viability of the MAC-T cells was assessed with a cell counting kit-8 (CCK-8 kit, Dojindo, Kumamoto, Japan). The cells were inoculated in 96-well plates (10^4^ cells per well) for 8 h, then treated with MNK or LPS at different levels. After 24 h of treatment, the cells were incubated with 10 μl of CCK-8 solution at 37°C for 1–4 h. The optical density of cells was measured at 450 nm with a microplate reader (TECAN, Safire, Austria). The ratio of optical density values of treated cells to untreated cells was used as cell viability.

### Intracellular ROS Measurement

The levels of intracellular ROS were measured by Dihydroethidium (Beyotime Biotech, Haimen, China). MAC-T were seeded in six-well plates at a density of 1×10^5^ cells/well and then treated with LPS or MNK for 24 h. After treatment, the cells were washed with phosphate buffer and then incubated with 0.5 µM DCFH-DA at 37°C for 30 min. After incubation, the cells were washed three times with serum-free DMEMF/12. After resuspension with appropriate DMEMF/12, the fluorescence intensity of the sample was observed under a fluorescence microscope (Olympus Co., Japan). The DHE fluorescence distribution of 10,000 cells was detected using a BD LSR flow cytometer (BD Biosciences, Franklin Lakes, NJ, United States) at an excitation wavelength of 488 nm and an emission wavelength of 535 nm.

### RNA Isolation and Quantitative Real-Time PCR (**qPCR**)

Total RNA was extracted from cells using a TRIZOL Regent Kit (Takara, Tokyo, Japan) according to the manufacturer’s instructions. And RNA concentration was detected by Nanodrop 2000 (Thermo, Massachusetts, United States). Then RNA was reverse transcribed to cDNA using a standard reverse transcription kit (Takara, Tokyo, Japan) according to the instructions. The primers were designed by the NCBI primer design tool, and are shown in Table 1 (synthesized in Comate Bioscience Co. Ltd., Changchun, China). qPCR was performed using the SYBR premix EX Taq (TaKaRa). Relative gene expression was normalized by GAPDH using the 2^−ΔΔCt^ method.

**TABLE 1:** Primers for qPCR.

Primer name	Primer sequence (5–3′)	Product length (bp)
*GAPDH*	Sense Primer: GTT​CAA​CGG​CAC​AGT​CAA​G	117
	Anti-sense Primer: TAC​TCA​GCA​CCA​GCA​TCA​C	
*IL-1*β	Sense Primer: CTG​TGG​AAC​CAA​TTT​CCG​GG	60
	Anti-sense Primer: AGG​CTT​GGT​GAA​AGG​ACT​TGA	
*IL-6*	Sense Primer: ATG​CTT​CCA​ATC​TGG​GTT​C	269
	Anti-sense Primer: TGA​GGA​TAA​TCT​TTG​CGT​TC	
*IL-8*	Sense Primer: GCT​GGC​TGT​TGC​TCT​CTT​G	126
	Anti-sense Primer: GGG​TGG​AAA​GGT​GTG​GAA​TG	
*TGFβ*	Sense Primer: AAC​CTG​TGT​TGC​TCT​CTC​GG	110
	Anti-sense Primer: GAG​GTA​GCG​CCA​GGA​ATT​GT	
*TNF-*α	Sense Primer: AGA​AGG​GAG​ATC​GCC​TCA​GT	62
	Anti-sense Primer: AGA​CTC​GGC​ATA​GTC​CAG​GT	
*NF-κB P65*	Sense Primer: TGG​CCC​CTA​TGT​GGA​GAT​CA	102
	Anti-sense Primer: CTC​CTC​TCT​CCA​GGG​ATG​CT	
*NF-κB P50*	Sense Primer: TTG​ACT​CAG​GTG​CAG​ACG​AC	101
	Anti-sense Primer: CCC​TGT​GGC​TTT​CCC​AGT​TT	
*HO-1*	Sense Primer: GGCAGCAAGGTGCAAGA	221
	Anti-sense Primer: GAA​GGA​AGC​CAG​CCA​AGA​G	

### Western Blot Analysis

The MAC-T cells were inoculated at a density of 1×10^5^ cells/well in 6-well plates and then treated with MNK or LPS for 24 h. There were three replicates of each group. Following treatment, cells were harvested and lysed in cold RIPA lysis buffer containing 1 mM PMSF (Beyotime Biotech, Haimen, China). The protein concentrations were assessed with a BCA protein assay kit (Beyotime, Shanghai, china). Equal amounts of protein were separated by 10% SDS-PAGE gels. Western blotting was performed as previously described (Wang et al., 2019).

### Statistical Analysis

All data were analyzed with one-way or two-way ANOVA with Dunnett’s multiple comparisons test by using SPSS 22.0 (StatSoft, United States). And the results are expressed as mean values ± standard deviation (**SD**). A value of *p* < 0.05 was regarded as statistically significant. *p* < 0.01 was considered extremely significant.

## Results

### Exogenous MNK Increased Cell Viability in LPS-Induced MAC-T

**FIGURE 2:**
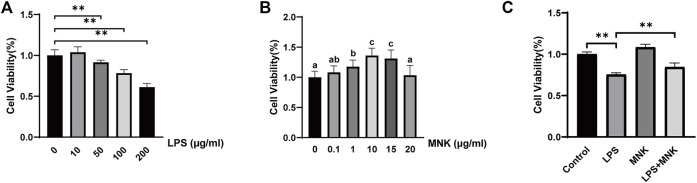
Effects of different concentrations of lipopolysaccharide (LPS) or 2-methyl nonyl ketone (MNK) on cell viability in MAC-T cells. **(A)** The effects of different concentrations of LPS (0, 10, 50, 100, and 200 μg/ml) for 24 h on the viability of MAC-T cells. **(B)** The effects of different concentrations of MNK (0, 0.1, 1, 10, 15 and 20 μg/ml) for 24 h on the viability of MAC-T cells. **(C)** The MAC-T cells were pretreated with MNK (10 μg/ml) for 4 h, followed by LPS (100 μg/ml) treatment for 24 h. The data are represented as mean ± SD. *N* = 6. **, *p* < 0.01.

Firstly, the effects of different concentrations of LPS on the viability of MAC-T cells were determined. As shown from Figure 2A, LPS decreased the viability of the MAC-T cells in a concentration-dependent manner. There was an extremely significant decrease at concentrations above 50 μg/ml. In addition, previous study has reported the inflammatory model *in vitro* with 100 μg/ml LPS (Sun et al., 2019). Thus 100 μg/ml was selected as the subsequent treatment concentration. Next, the effects of MNK on the cell viability at 24 h were examined (Figure 2B). The results shown that no significant difference was observed at 0.1 or 20 μg/ml. However, there was an extremely significant increase at concentrations of 1, 10, and 15 μg/ml. The cell viability was highest at 10 μg/ml MNK. Then, the effects of MNK on the cell viability with or without LPS at 24 h were examined. It was shown that decreased cell viability induced by LPS was reversed by MNK (Figure 2C).

### Exogenous MNK Induced ROS Production in LPS-Induced MAC-T

**FIGURE 3:**
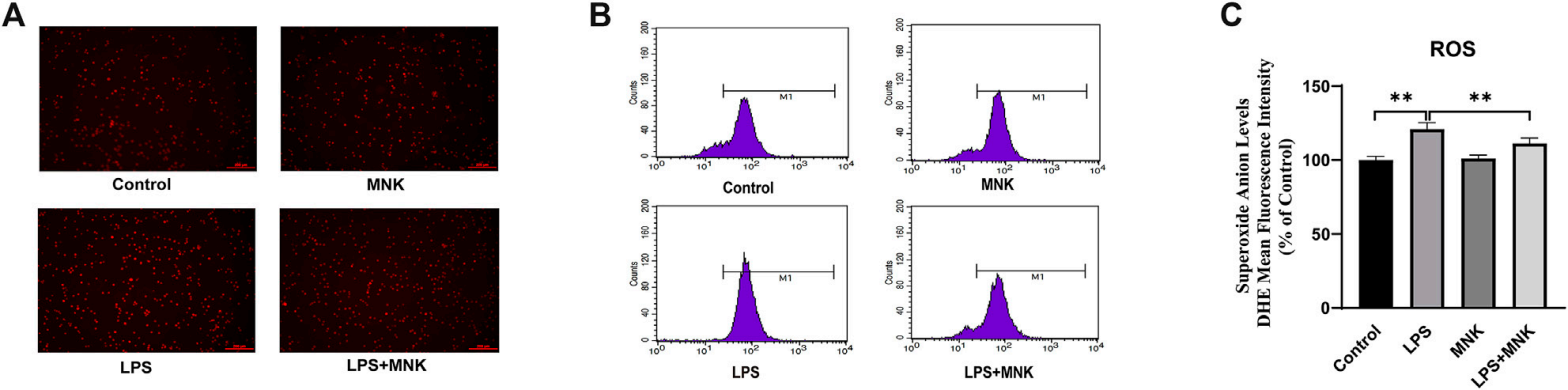
Effects of lipopolysaccharide (LPS, 100 μg/ml) or 2-methyl nonyl ketone (MNK, 10 μg/ml) on reactive oxygen species (ROS) production in MAC-T cells. **(A)** The higher the level of superoxide anion, the stronger the red fluorescence. The intensity of red fluorescence was stronger in the LPS group compared with the control group. And intensity in LPS plus MNK group was weaker than in the LPS group. **(B)** The fluorescence distribution of sample. **(C)** Superoxide anion levels. The data are represented as mean ± SD. *N* = 3. **, *p* < 0.01.

To verify whether the mechanism by which MNK exerts its protective effect is related to ROS, ROS in cells were assayed with Dihydroethidium. In Figure 3A, the stronger fluorescence intensity in the LPS group indicated a higher level of intracellular ROS compared to the control group. In contrast, the fluorescence intensity of the LPS plus MNK group was weaker than LPS group, indicating a lower level of intracellular ROS. Meanwhile, the fluorescence intensity was quantified using a BD LSR flow cytometer. As shown in Figure 3C, ROS production of cells treated with 100 μg/ml LPS was significantly increased compared to the control group. However, ROS production was significantly reduced in the LPS plus MNK group compared to the LPS group.

### Effect of MNK on mRNA Levels of Inflammatory Factors Induced by LPS in MAC-T Cells

**FIGURE 4:**
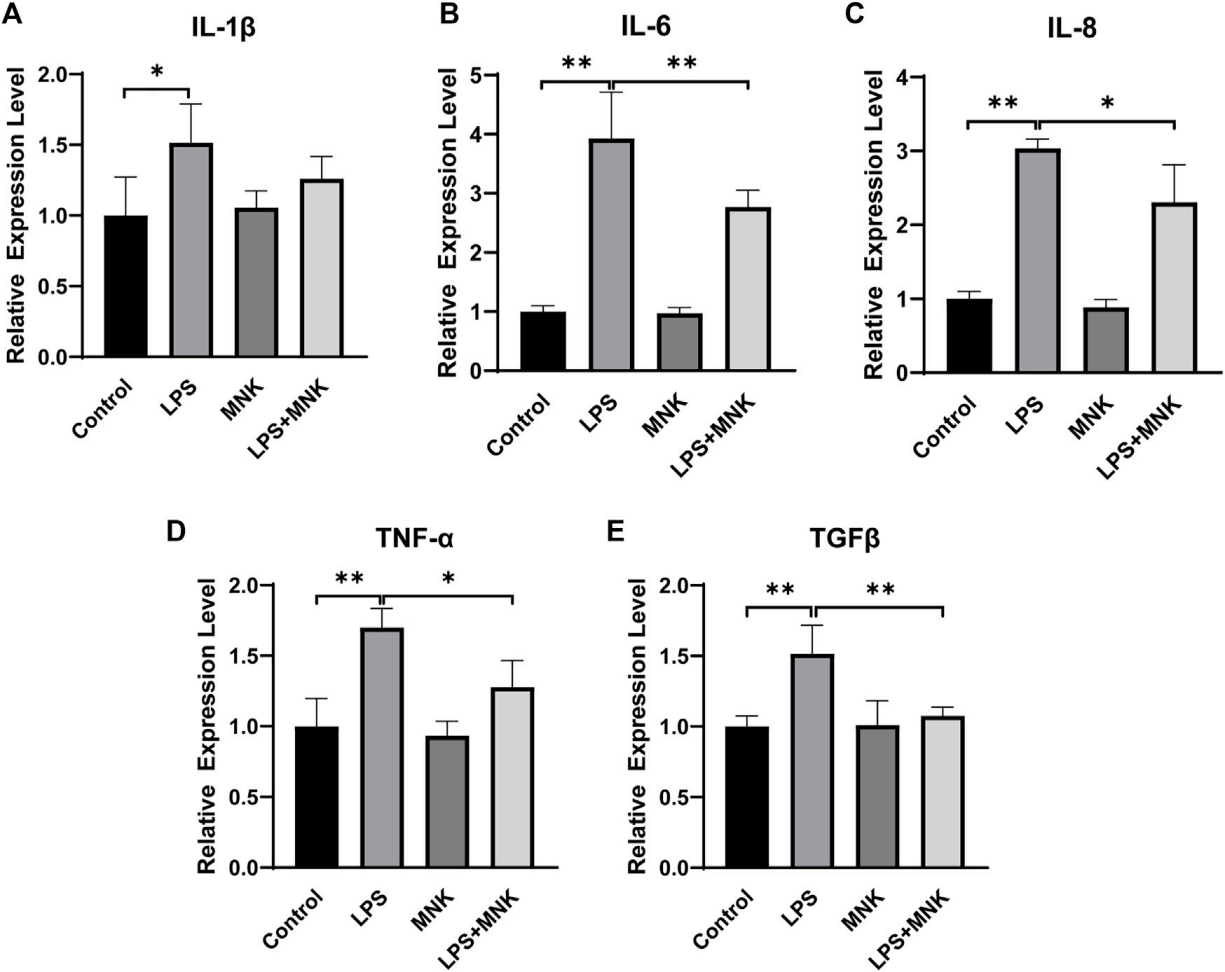
Effect of 2-methyl nonyl ketone (MNK, 10 μg/ml) on the relative mRNA expression levels of inflammation-related gene in lipopolysaccharide (LPS, 100 μg/ml)-induced MAC-T cells. **(A)** *Interleukin* (*IL*)*-1β*. **(B)** *IL-6*. **(C)** *IL-8*. **(D)** *Tumor necrosis factor-α* (*TNF-*α). **(E)** *Transforming growth factor beta* ***(TGFβ)***. The data are shown as mean ± SD. *N* = 3. *, *p* < 0.05; **, *p* < 0.01.

To further analyze the effect of MNK on LPS-induced inflammation in MAC-T cells, the mRNA expression levels of inflammation cytokines were examined by qPCR. As shown in Figure 4, it is apparent that LPS significantly increased the mRNA expression of *IL-1β*, *IL-6*, *IL-8*, *TNF-α*, and *TGFβ*. Compared with the LPS group, the mRNA expression of *IL-1β*, *IL-6*, *IL-8*, *TNF-α*, and *TGFβ* was significantly decreased in the LPS plus MNK group.

### Effect of MNK on LPS Induced Inflammation-Associated Protein Expression

**FIGURE 5:**
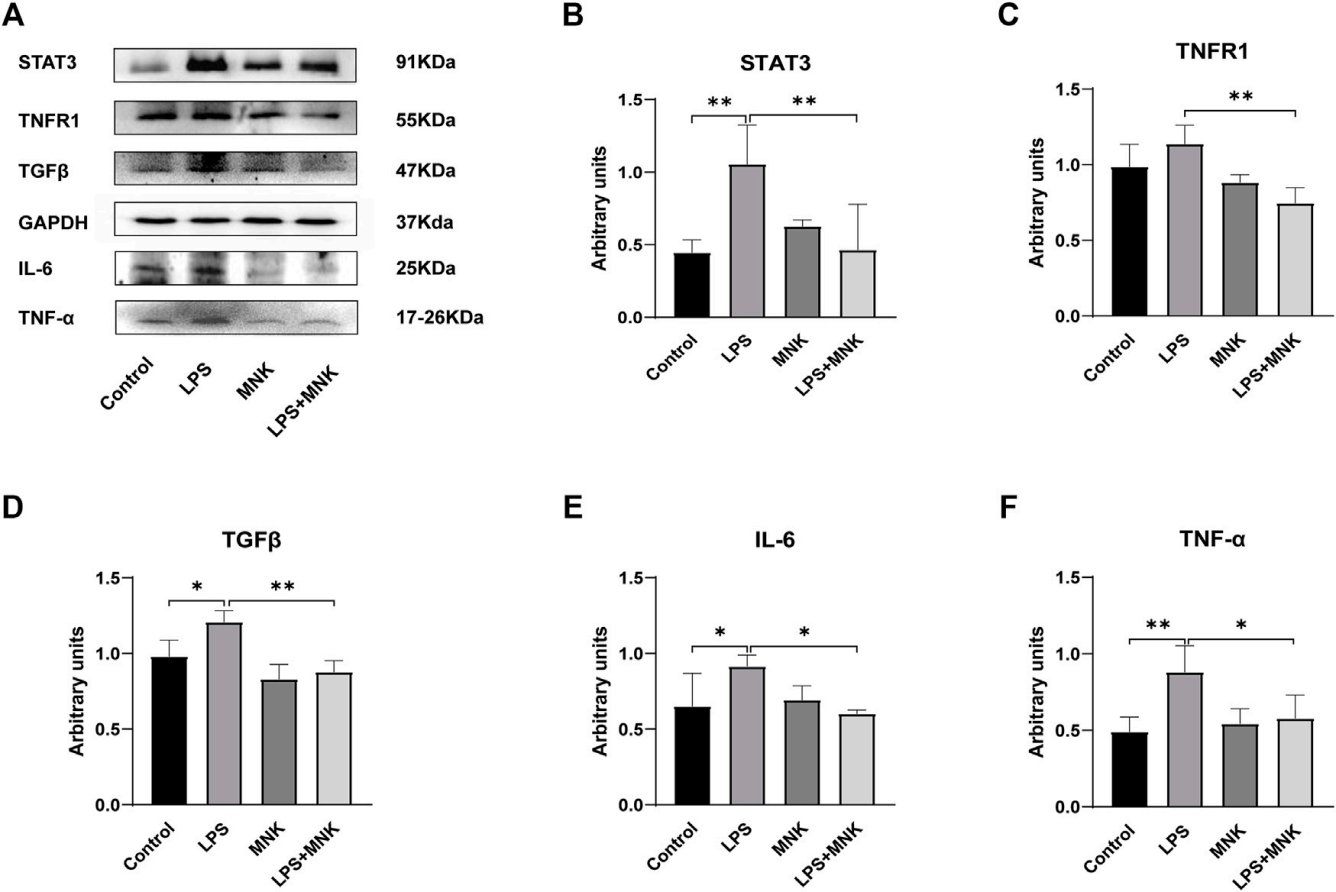
Effect of 2-methyl nonyl ketone (MNK, 10 μg/ml) on the expression levels of inflammation-related proteins in lipopolysaccharide (LPS, 100 μg/ml)-induced MAC-T cells. **(A)** The expression of the inflammation-related protein was measured using Western blot. **(B)** Relative levels of signal transducer and activator of transcription (STAT3) were analyzed by grey scanning. **(C)** TNF-receptor-associated complex I (TNFR1). **(D)** Transforming growth factor beta (TGFβ). **(E)** Interleukin (IL)-6. **(F)** Tumor necrosis factor-α (TNF-α). GAPDH was used as an internal reference for Western blotting analysis. The data are shown as mean ± SD. *N* = 3. *, *p* < 0.05; **, *p* < 0.01.

The effect of MNK on the expression of inflammation-related proteins in LPS-induced MAC-T cells was verified by Western blotting. As shown in Figure 5, LPS significantly upregulated the protein expression of signal transducer and activator of transcription (**STAT3**), IL-6,TNF-α, and TGFβ, compared with the control group. In addition, MNK inhibited LPS-induced protein expression of STAT3, IL-6, TNF-α, TGFβ and TNF-receptor-associated complex I (**TNFR1**).

### Effect of MNK on TLR4 Signaling Pathway Induced by LPS in MAC-T Cells

**FIGURE 6:**
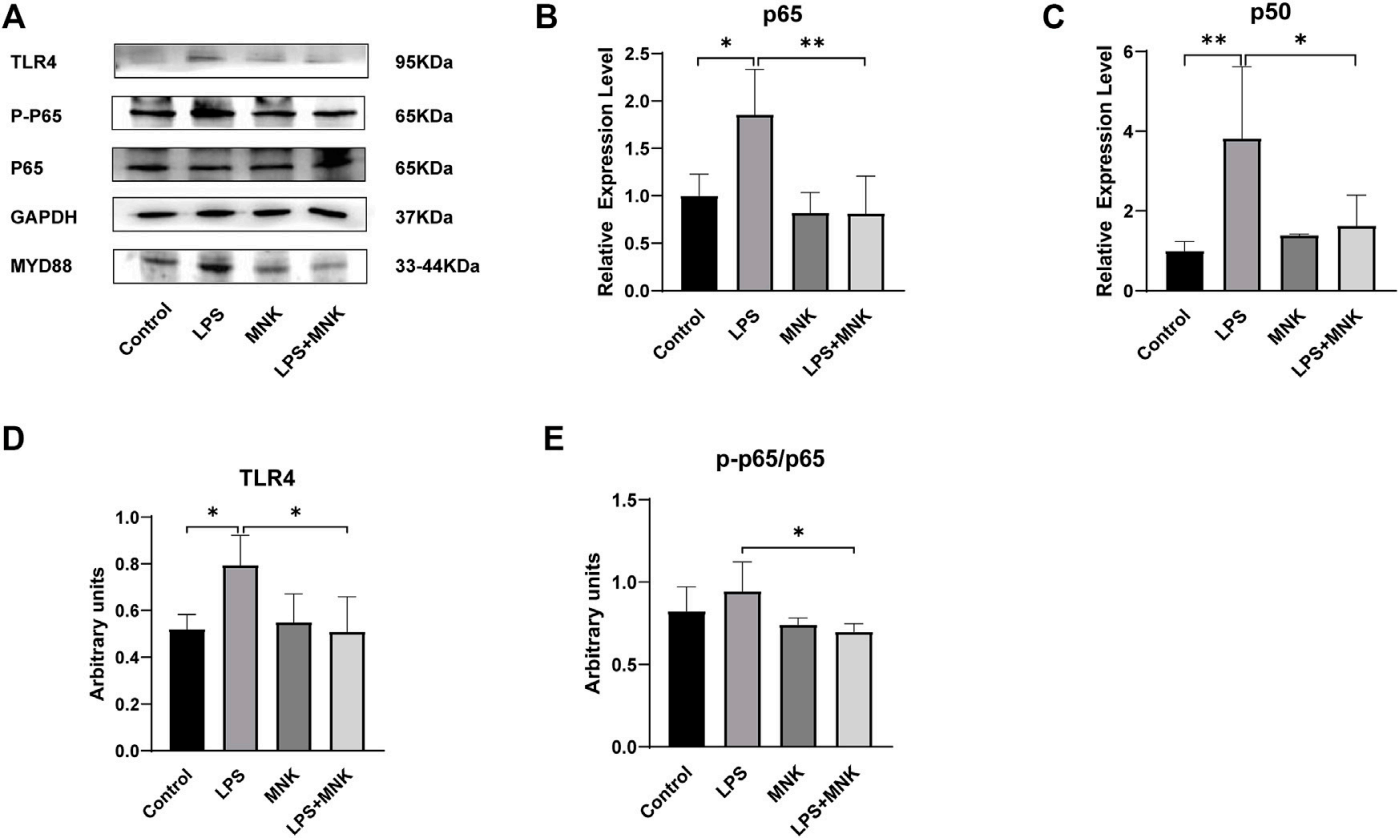
The effects of 2-methyl nonyl ketone (MNK, 10 μg/ml) on the levels of Toll-like receptor 4-nuclear factor-κB (TLR4-NF-κB) signaling pathway induced by lipopolysaccharide (LPS, 100 μg/ml) in MAC-T cells. **(A)** The expression of the TLR4 signaling pathway was measured using Western blot. **(B)** *p65*. **(C)** *p50*. **(D)** TLR4. **(E)** p-p65/p65. GAPDH was used as an internal reference for Western blotting analysis. The data are shown as mean ± SD. *N* = 3. *, *p* < 0.05; **, *p* < 0.01.

TLR4, myeloid differentiation factor 88 (**MYD88**), p-p65 and p65 protein expression levels were measured by Western blot analysis to investigate whether the protective effect of exogenous MNK was related to the TLR4 signaling pathway. As shown in Figure 6, the mRNA levels of *p65* and *p50* were significantly increased after treatment with LPS, but were reversed by MNK (10μg/ml) at 24 h in MAC-T cells. In addition, MNK inhibited LPS-induced TLR4, p-p65/p65 protein expression.

### Effect of MNK on Nrf2 Signaling Pathway Induced by LPS in MAC-T Cells

**FIGURE 7:**
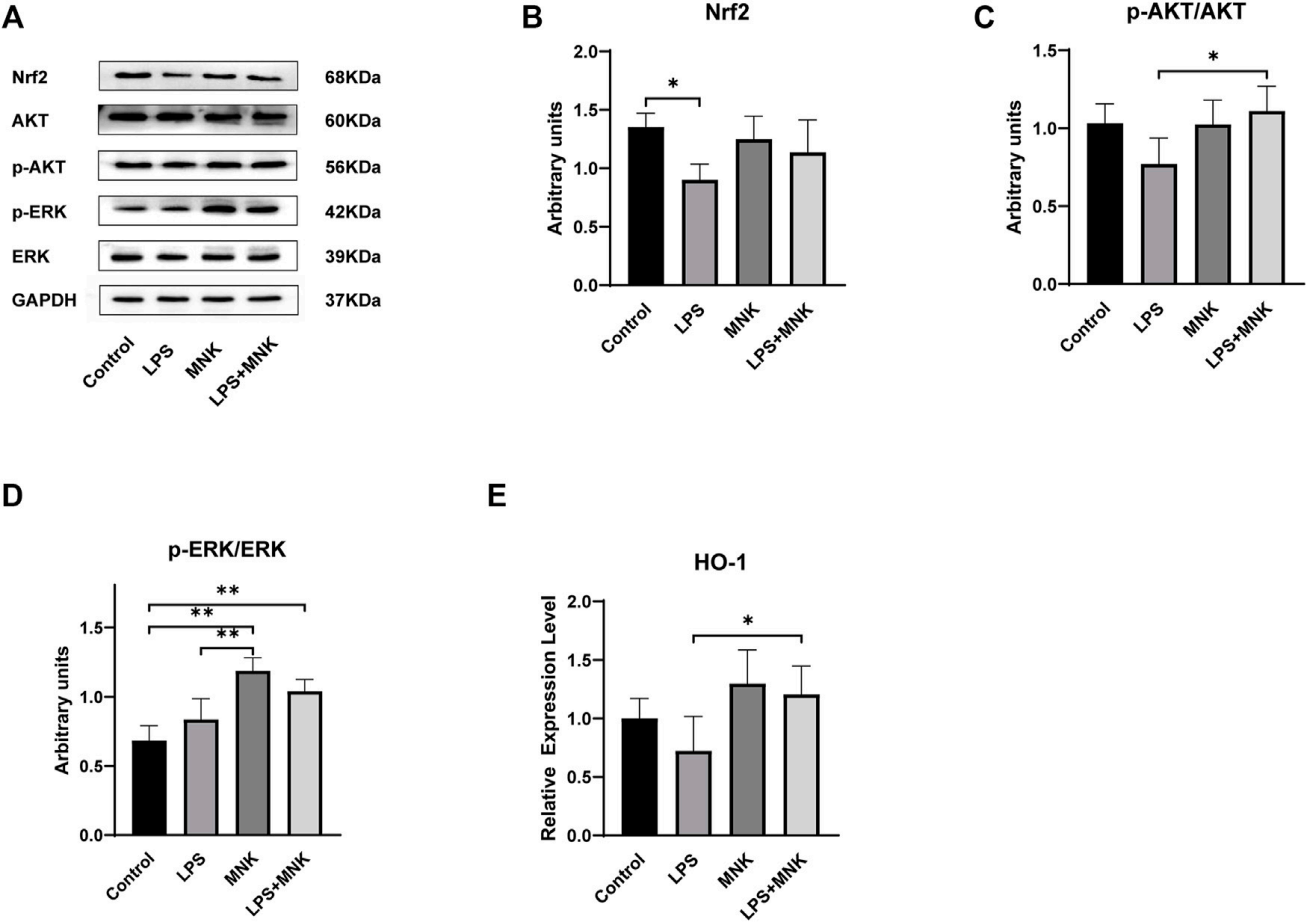
The effects of 2-methyl nonyl ketone (MNK, 10 μg/ml) on the levels of Nuclear factor erythroid 2-related factor 2 (Nrf2) signaling pathway induced by lipopolysaccharide (LPS, 100 μg/ml) in MAC-T cells. **(A)** The expression of the Nrf2 signaling pathway was measured using Western blot. **(B)** Nrf2. **(C)** p-AKT/AKT. **(D)** p-extracellular signal-regulated kinase (ERK)/ERK. **(E)** The mRNA relative expression levels of *Heme Oxygenaseoxygenase-1* (*HO-1*). GAPDH was used as an internal reference for Western blotting analysis. The data are shown as mean ± SD. *N* = 3. *, *p* < 0.05; **, *p* < 0.01.

The protective effect of MNK on LPS-induced MAC-T cell injury was investigated by Western blotting to determine whether it was related to the Nrf2 signaling pathway. The results showed that Nrf2 protein expression was significantly decreased in the LPS group compared to the control group. Nrf2 protein expression in the LPS + MNK groups was not significantly different from the control group. MNK reversed the reduction of Nrf2 protein expression induced by LPS (Figure 7B). The protein expression levels of p-AKT, AKT, p-ERK and ERK were further analyzed to determine whether the effect of MNK increasing the Nrf2 expression was related to the AKT and ERK pathways. As shown in Figure 7C, the p-AKT/AKT protein levels were significantly increased in the LPS plus MNK group compared with the LPS group. Compared with the control group, the addition of MNK resulted in a significant increase in p-ERK/ERK. In addition, the mRNA expression level of *HO-1* was significantly increased in the LPS plus MNK groups compared to the LPS group.

## Discussion

Microbial infection is the main cause of mastitis (Graber and Bodmer, 2019). Pathogens invade the mammary gland and release macromolecules such as LPS to stimulate the immune response and cause mastitis (Cheng et al., 2019). However, antibiotics for mastitis cause antibiotic residues in milk, which damage human health (Anika et al., 2019). The development of more effective agents alternative to antibiotics for mastitis treatment is urgently needed. *H. cordata* has high nutritional and medicinal value and can be used in the treatment of inflammation (Shingnaisui et al., 2018) and has promising market development prospects as an alternative to antibiotics. However, the pharmacological effects and mechanisms for each component of *H. cordata* have not been fully investigated due to its complex composition. This is not conducive to its quality control and dose determination, which limits the further application of *H. cordata*. MNK is the most abundant component of the volatile oil from the hydrodistillation extraction of *H. cordata* (Lou et al., 2019). And there have been some researches showing that MNK reduces adverse symptoms by inhibiting inflammatory responses in mice (Chen et al., 2014). However, the anti-inflammatory effect of MNK on bovine mammary epithelial cells and its molecular mechanism have not been investigated. LPS induces cell death by stimulating an increase in inflammatory cytokines and ROS (Qiu et al., 2019). In this study, the inflammatory model of mastitis in dairy cows was simulated using LPS-treated MAC-T cells to investigate the protective effect of adding MNK against LPS-induced cell damage. This work will contribute to the understanding of the anti-inflammatory effects of *H. cordata* and also provide a basis for the development of natural and effective drug candidates.

Bacterial infection of the mammary gland is accompanied by the release of endotoxins, proteoglycans and enzymes (Wang et al., 2021). This stimulates the immune response, leading to an increase in the number of somatic cells in the milk and the level of circulating endotoxin, which leads to an increase in the expression of pro-inflammatory cytokines and chemokines (Ashraf and Imran, 2020). IL-6, IL-8, and TNF-α are important pro-inflammatory cytokines involved in mastitis (Zhu et al., 2007; Imam et al., 2021; Liu et al., 2021). In the present study, LPS significantly decreased the cell viability of MAC-T cells with a dose-dependent effect, indicating that LPS causes cell damage to MAC-T cells. Exogenous addition of MNK reversed the LPS-induced decrease in cell viability. This suggests that MNK has a protective effect on LPS-induced cell injury. Next, 100 μg/ml LPS was selected to establish an inflammation model to simulate mastitis, and the expression levels of mRNA and protein of inflammation-related factors were examined. The results showed that MNK significantly inhibited LPS-induced upregulation of mRNA levels of *IL-1*β, *IL-6*, *IL-8*, *TNF-*α, and *TGFβ* and protein levels of STAT3, IL-6, TNFα, TNFR1 and TGFβ. STAT3 is a downstream signaling regulatory molecule of IL-6 (Latourte et al., 2017). TNFR1 is a binding receptor for TNF-α, and TNF-α induces necroptosis in cells through TNFR1 (Amin et al., 2018). It implies that MNK acts cytoprotectively by reducing the production of pro-inflammatory cytokines and inhibiting their binding to receptors.

TLR4 is essential for LPS-stimulated inflammatory factor release (Bhattarai et al., 2018). LPS forms a dimeric complex with TLR4, which then activates downstream signaling pathways via MYD88 (Song et al., 2014). NF-κB plays a key role in promoting inflammatory cytokine expression (Chen et al., 2018). To further investigate the mechanism by which MNK exerts the anti-inflammatory effects, this study examined the effects of MNK on LPS-induced TLR4, MYD88, and NF-κB signaling pathways. In this study, MNK inhibited the expression of TLR4, MYD88 (Supplementary Material S1), NF-κB and LPS-induced phosphorylation of p65. This demonstrates that MNK has an anti-inflammatory effect in the LPS-induced mastitis model by inhibiting the TLR4-NF-κB signaling pathway.

LPS disrupts the homeostasis of the redox system in bovine mammary epithelial cells, leading to increased levels of ROS and oxidative stress. Oxidative stress leads to mitochondrial dysfunction, abnormal protein synthesis, and induction of cellular autophagy to cause cellular damage (Ju et al., 2021). The results of this study showed that MNK pretreatment reduced the LPS-induced elevation of ROS levels. This indicates that MNK could reduce LPS-induced oxidative stress in MAC-T cells. Nrf2 is an important regulator that induces the expression of antioxidant enzymes (Zhou et al., 2021). Previous studies have shown that activation of AKT and ERK pathways can increase Nrf2 nuclear translocation thereby inhibiting the overproduction of ROS (Wang et al., 2019; Cao et al., 2021). The results of the present study are consistent with the findings that MNK activated AKT and ERK pathways and increased Nrf2 protein expression, which in turn promoted *HO-1* expression and attenuated LPS-induced oxidative stress. Excessive ROS triggers inflammation (Li et al., 2021). MNK may also inhibit the activation of NF-κB pathway by reducing ROS levels through Nrf2 pathways, which in turn exerts anti-inflammatory effects.

**FIGURE 8:**
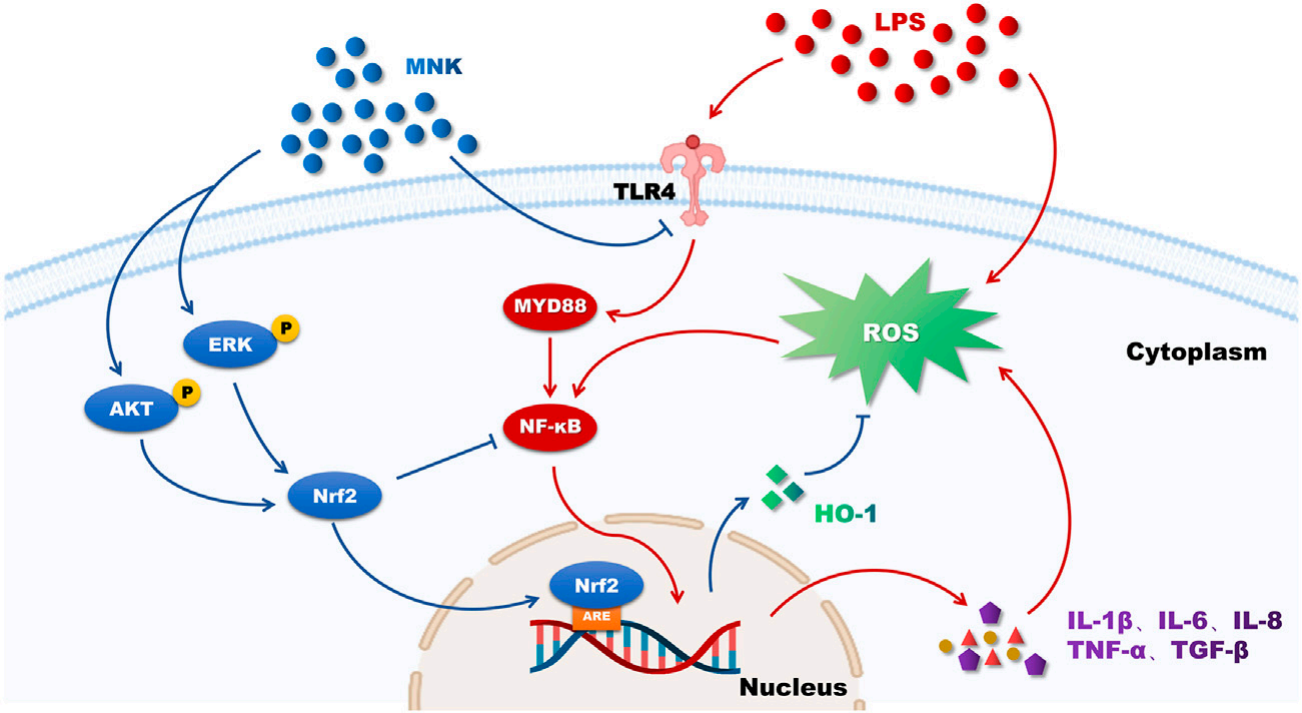
The putative mechanism of 2-methyl nonyl ketone (MNK) attenuating the inflammatory response and oxidative stress of MAC-T cells induced by lipopolysaccharide (LPS). Red arrows indicate the effects of LPS on MAC-T cells and blue arrows indicate the protective effects of MNK.

In conclusion, MNK has a protective effect on LPS-induced mammary epithelial cell damage in cows (Figure 8). This suggests that MNK may serve as an effective candidate for the treatment of mastitis in dairy cows, but it needs to be verified by *in vivo* experiments in dairy cows. This work will provide a theoretical basis for the study of the pharmacodynamic mechanism of *H. cordata* and its application in the prevention and treatment work of mastitis in dairy cows.

## Conclusion

In this study, MNK was found to have a protective effect on the inflammatory response and oxidative stress in mammary epithelial cells of dairy cows. Pretreatment with MNK reduced the LPS-induced inflammatory response by inhibiting the TLR4-NF-κB signaling pathway and pro-inflammatory factors receptor recognition. In addition, MNK may activate Nrf2 through AKT and ERK pathways, which in turn inhibits ROS production. This facilitates the protection of MAC-T cells from LPS-induced inflammatory response and oxidative damage.

## Data Availability

The original contributions presented in the study are included in the article/Supplementary Material, further inquiries can be directed to the corresponding authors.

## References

[B1] Aghamohammadi M. Haine D. Kelton D. F. Barkema H. W. Hogeveen H. Keefe G. P. (2018). Herd-Level Mastitis-Associated Costs on Canadian Dairy Farms. Front. Vet. Sci. 5, 100. 10.3389/fvets.2018.00100 29868620 PMC5961536

[B2] Amin P. Florez M. Najafov A. Pan H. Geng J. Ofengeim D. (2018). Regulation of a Distinct Activated RIPK1 Intermediate Bridging Complex I and Complex II in TNFα-Mediated Apoptosis. Proc. Natl. Acad. Sci. USA 115, E5944–E5953. 10.1073/pnas.1806973115 29891719 PMC6042106

[B3] Anika T. Noman Z. Ferdous M. Khan S. Mukta M. Islam M. (2019). Time Dependent Screening of Antibiotic Residues in Milk of Antibiotics Treated Cows. J. Adv. Vet. Anim. Res. 6, 516–520. 10.5455/javar.2019.f376 31819880 PMC6882713

[B4] Ashraf A. Imran M. (2020). Causes, Types, Etiological Agents, Prevalence, Diagnosis, Treatment, Prevention, Effects on Human Health and Future Aspects of Bovine Mastitis. Anim. Health Res. Rev. 21, 36–49. 10.1017/S1466252319000094 32051050

[B5] Bhattarai D. Worku T. Dad R. Rehman Z. U. Gong X. Zhang S. (2018). Mechanism of Pattern Recognition Receptors (PRRs) and Host Pathogen Interplay in Bovine Mastitis. Microb. Pathogenesis 120, 64–70. 10.1016/j.micpath.2018.04.010 29635052

[B6] Cao J. Lu M. Yan W. Li L. Ma H. (2021). Dehydroepiandrosterone Alleviates Intestinal Inflammatory Damage via GPR30-Mediated Nrf2 Activation and NLRP3 Inflammasome Inhibition in Colitis Mice. Free Radic. Biol. Med. 172, 386–402. 10.1016/j.freeradbiomed.2021.06.025 34182071

[B7] Chen J. Wang W. Shi C. Fang J. (2014). A Comparative Study of Sodium Houttuyfonate and 2-undecanone for Their *In Vitro* and *In Vivo* Anti-inflammatory Activities and Stabilities. Ijms 15, 22978–22994. 10.3390/ijms151222978 25514406 PMC4284749

[B8] Chen X. Zheng X. Zhang M. Yin H. Jiang K. Wu H. (2018). Nuciferine Alleviates LPS-Induced Mastitis in Mice via Suppressing the TLR4-NF-Κb Signaling Pathway. Inflamm. Res. 67, 903–911. 10.1007/s00011-018-1183-2 30145653

[B9] Cheng W. N. Jeong H. J. Seo H. G. Han S. G. (2019). Moringa Extract Attenuates Inflammatory Responses and Increases Gene Expression of Casein in Bovine Mammary Epithelial Cells. Animals 9, 391. 10.3390/ani9070391 PMC6680921 31248033

[B10] Chou S.-C. Su C.-R. Ku Y.-C. Wu T.-S. (2009). The Constituents and Their Bioactivities of Houttuynia Cordata. Chem. Pharm. Bull. 57, 1227–1230. 10.1248/cpb.57.1227 19881272

[B11] Graber H. Bodmer M. (2019). *Staphylococcus aureus* and its Genotypes as a Mastitis Pathogen in Dairy Cattles - a Review. Sat 161, 611–617. 10.17236/sat00223 31586923

[B12] Guo W. Liu B. Yin Y. Kan X. Gong Q. Li Y. (2019). Licochalcone A Protects the Blood-Milk Barrier Integrity and Relieves the Inflammatory Response in LPS-Induced Mastitis. Front. Immunol. 10, 287. 10.3389/fimmu.2019.00287 30858849 PMC6398509

[B13] Hadrich J. C. Wolf C. A. Lombard J. Dolak T. M. (2018). Estimating Milk Yield and Value Losses from Increased Somatic Cell Count on US Dairy Farms. J. Dairy Sci. 101, 3588–3596. 10.3168/jds.2017-13840 29398029

[B14] Hemalatha S. Kumar M. Prasad S. (2014). A Current Update on the Phytopharmacological Aspects of Houttuynia Cordata Thunb. Phcog Rev. 8, 22–35. 10.4103/0973-7847.125525 24600193 PMC3931198

[B15] Imam B. H. Oladejo A. O. Wu X. Yang J. Ma X. Shen W. (2021). Anti-Inflammatory and Antibacterial Potential of Qicao Rukang Powder in Bovine Subclinical Mastitis. Evidence-Based Complement. Altern. Med. 2021, 1–10. 10.1155/2021/2148186 PMC8416365 34484387

[B16] Ju L. Zhang J. Wang F. Zhu D. Pei T. He Z. (2021). Chemical Profiling of Houttuynia Cordata Thunb. By UPLC-Q-TOF-MS and Analysis of its Antioxidant Activity in C2C12 Cells. J. Pharm. Biomed. Anal. 204, 114271. 10.1016/j.jpba.2021.114271 34325249

[B17] Klaas I. C. Zadoks R. N. (2018). An Update on Environmental Mastitis: Challenging Perceptions. TRANSBOUND EMERG. DIS. 65 (Suppl. 1), 166–185. 10.1111/tbed.12704 29083115

[B18] Latourte A. Cherifi C. Maillet J. Ea H.-K. Bouaziz W. Funck-Brentano T. (2017). Systemic Inhibition of IL-6/Stat3 Signalling Protects against Experimental Osteoarthritis. ANN. RHEUM. DIS. 76, 748–755. 10.1136/annrheumdis-2016-209757 27789465

[B19] Li D. Liu J.-P. Han X. Wang Y.-F. Wang C.-H. Li Z. (2017). Chemical Constituents of the Whole Plants of Houttuynia Cordata. Chem. Nat. Compd. 53, 365–367. 10.1007/s10600-017-1991-6

[B20] Li L. Cheng S. Q. Guo W. Cai Z. Y. Sun Y. Q. Huang X. X. (2021). Oridonin Prevents Oxidative Stress‐induced Endothelial Injury via Promoting Nrf‐2 Pathway in Ischaemic Stroke. J. CELL MOL. MED. 25, 9753–9766. 10.1111/jcmm.16923 34514714 PMC8505855

[B21] Liu L. Lu H. Loor J. J. Aboragah A. Du X. He J. (2021). Sirtuin 3 Inhibits Nuclear Factor-Κb Signaling Activated by a Fatty Acid challenge in Bovine Mammary Epithelial Cells. J. Dairy Sci. 104, 12871–12880. 10.3168/jds.2021-20536 34482974

[B22] Lou Y. Guo Z. Zhu Y. Kong M. Zhang R. Lu L. (2019). Houttuynia Cordata Thunb. And its Bioactive Compound 2-undecanone Significantly Suppress Benzo(a)pyrene-Induced Lung Tumorigenesis by Activating the Nrf2-HO-1/nqo-1 Signaling Pathway. J. Exp. Clin. Cancer Res. 38, 242. 10.1186/s13046-019-1255-3 31174565 PMC6556055

[B23] Puerto M. A. Shepley E. Cue R. I. Warner D. Dubuc J. Vasseur E. (2021). The Hidden Cost of Disease: I. Impact of the First Incidence of Mastitis on Production and Economic Indicators of Primiparous Dairy Cows. J. Dairy Sci. 104, 7932–7943. 10.3168/jds.2020-19584 33865582

[B24] Qiu Z. He Y. Ming H. Lei S. Leng Y. Xia Z.-y. (2019). Lipopolysaccharide (LPS) Aggravates High Glucose- and Hypoxia/Reoxygenation-Induced Injury through Activating ROS-dependent NLRP3 Inflammasome-Mediated Pyroptosis in H9C2 Cardiomyocytes. J. Diabetes Res. 2019, 1–12. 10.1155/2019/8151836 PMC6398034 30911553

[B25] Shingnaisui K. Dey T. Manna P. Kalita J. (2018). Therapeutic Potentials of Houttuynia Cordata Thunb. Against Inflammation and Oxidative Stress: A Review. J. Ethnopharmacology 220, 35–43. 10.1016/j.jep.2018.03.038 PMC7127360 29605674

[B26] Song X. Zhang W. Wang T. Jiang H. Zhang Z. Fu Y. (2014). Geniposide Plays an Anti-inflammatory Role via Regulating TLR4 and Downstream Signaling Pathways in Lipopolysaccharide-Induced Mastitis in Mice. INFLAMMATION 37, 1588–1598. 10.1007/s10753-014-9885-2 24771071

[B27] Subhawa S. Naiki-Ito A. Kato H. Naiki T. Komura M. Nagano-Matsuo A. (2021). Suppressive Effect and Molecular Mechanism of Houttuynia Cordata Thunb. Extract against Prostate Carcinogenesis and Castration-Resistant Prostate Cancer. Cancers 13, 3403. 10.3390/cancers13143403 34298624 PMC8306559

[B28] Sun L. Chen L. Wang F. Zheng X. Yuan C. Niu Q. (2019). Exogenous Hydrogen Sulfide Prevents Lipopolysaccharide-Induced Inflammation by Blocking the TLR4/NF-Κb Pathway in MAC-T Cells. GENE 710, 114–121. 10.1016/j.gene.2019.05.033 31153885

[B29] Wang F. Zhao Y. Chen S. Chen L. Sun L. Cao M. (2019). Astragaloside IV Alleviates Ammonia-Induced Apoptosis and Oxidative Stress in Bovine Mammary Epithelial Cells. Ijms 20, 600. 10.3390/ijms20030600 PMC6386910 30704086

[B30] Wang N. Zhou C. Basang W. Zhu Y. Wang X. Li C. (2021). Mechanisms by Which Mastitis Affects Reproduction in Dairy Cow: A Review. Reprod. Dom Anim. 56, 1165–1175. 10.1111/rda.13953 34008236

[B31] Wu X. Li J. Wang S. Jiang L. Sun X. Liu X. (2021). 2-Undecanone Protects against Fine Particle-Induced Kidney Inflammation via Inducing Mitophagy. J. Agric. Food Chem. 69, 5206–5215. 10.1021/acs.jafc.1c01305 33877841

[B32] Zhou X. Afzal S. Zheng Y.-F. Münch G. Li C. G. (2021). Synergistic Protective Effect of Curcumin and Resveratrol against Oxidative Stress in Endothelial EAhy926 Cells. Evidence-Based Complement. Altern. Med. 2021, 1–13. 10.1155/2021/2661025 PMC8434903 34518768

[B33] Zhu Y. Fossum C. Berg M. Magnusson U. (2007). Morphometric Analysis of Proinflammatory Cytokines in Mammary Glands of Sows Suggests an Association between Clinical Mastitis and Local Production of IL-1beta, IL-6 and TNF-Alpha. Vet. Res. 38, 871–882. 10.1051/vetres:2007035 17903420

