# Supplementary figures and images for: 2-Methyl Nonyl Ketone From Houttuynia Cordata Thunb Alleviates LPS-Induced Inflammatory Response and Oxidative Stress in Bovine Mammary Epithelial Cells
Source: Front Chem. 2022 Jan 31;9:793475. doi: 10.3389/fchem.2021.793475 (PMC8842123; doi:10.3389/fchem.2021.793475)

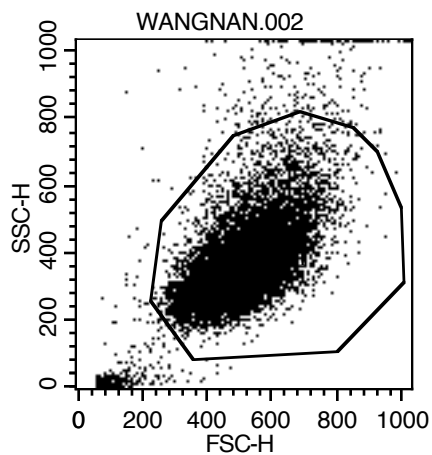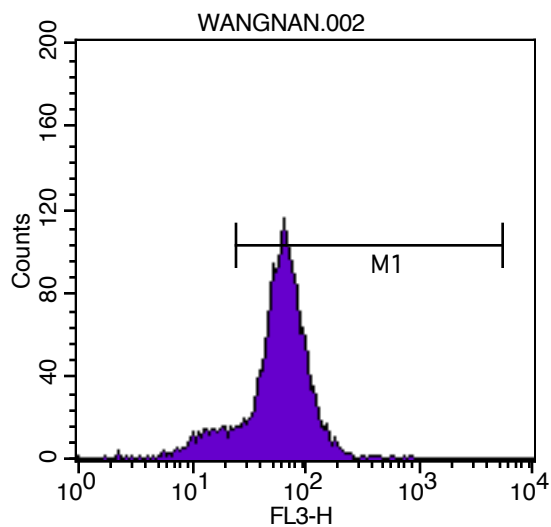

Gate: G1  
Total Events: 10598  
Gated Events: 1000  
X Parameter: FL3-H

| Marker | Events | % Gated | Mean  |
|--------|--------|---------|-------|
| All    | 10000  | 100.00  | 63.33 |
| M1     | 8990   | 89.90   | 68.73 |

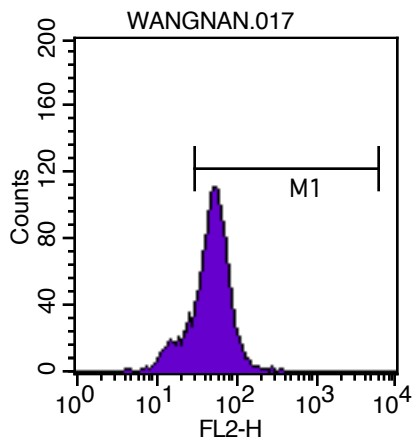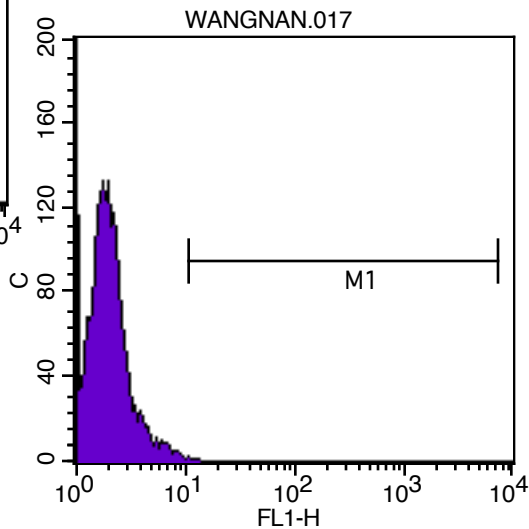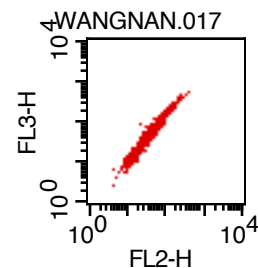

Supplement: Supplementary file 1 [file DataSheet1.ZIP › ROS/flow cytometry data/Controul/2.pdf]

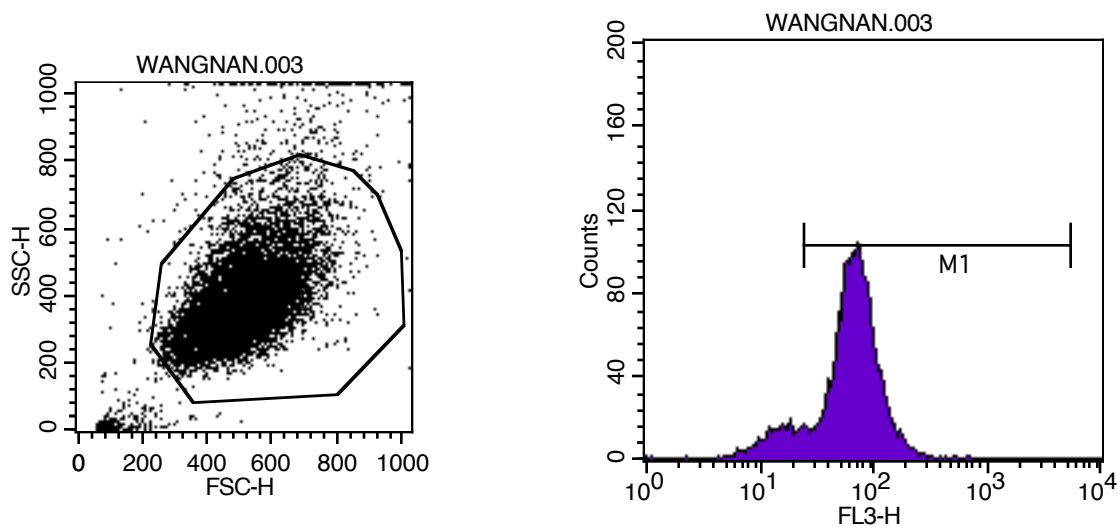

Gate: G1  
Total Events: 10653

Gated Events: 1000  
X Parameter: FL3-H

| Marker | Events | % Gated | Mean  |
|--------|--------|---------|-------|
| All    | 10000  | 100.00  | 66.49 |
| M1     | 8887   | 88.87   | 72.89 |

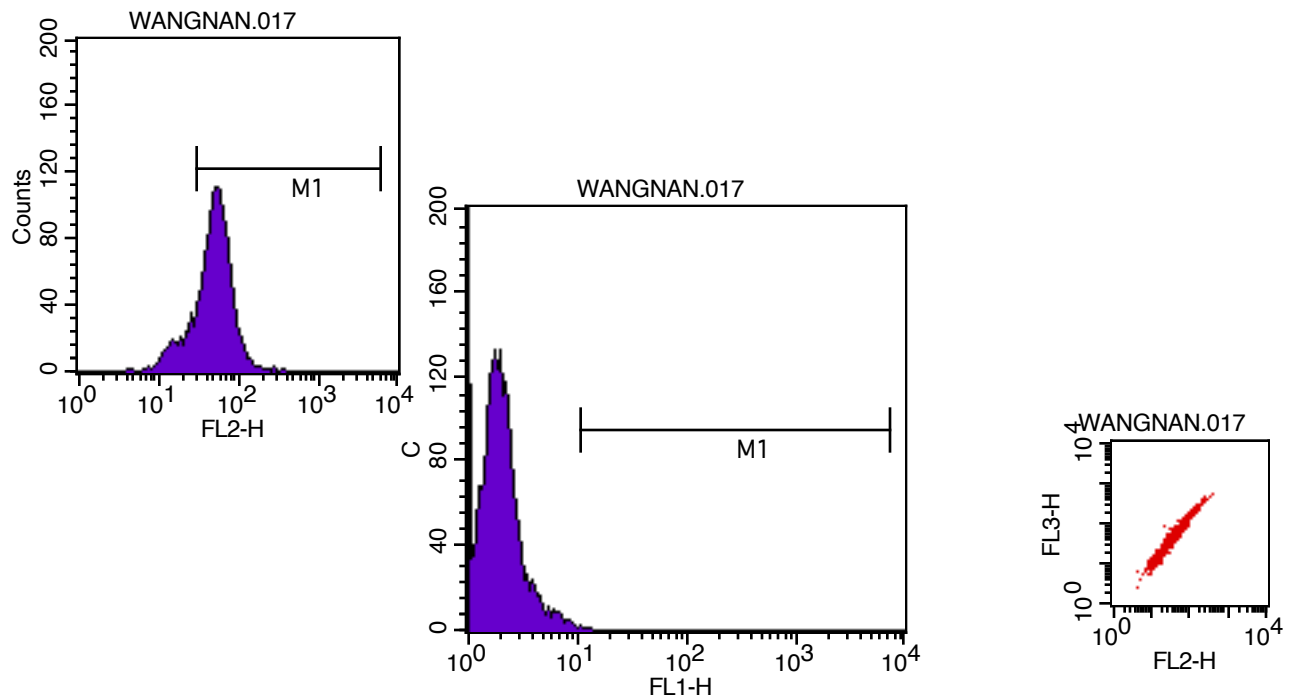

Supplement: Supplementary file 1 [file DataSheet1.ZIP › ROS/flow cytometry data/Controul/3.pdf]

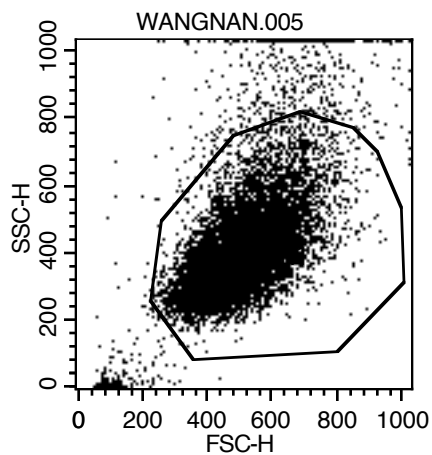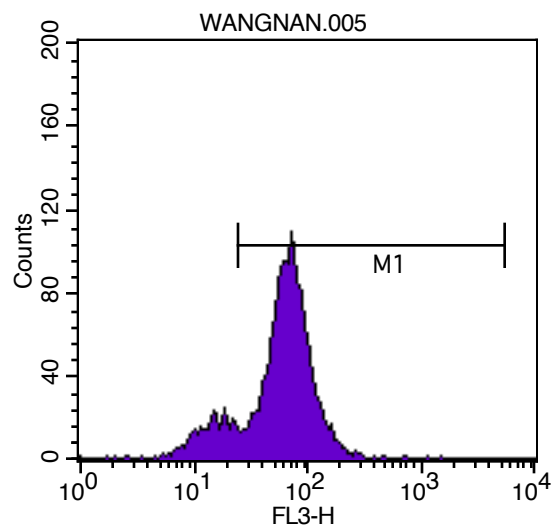

Gate: G1  
 Total Events: 10744  
 Gated Events: 1000  
 X Parameter: FL3-H

| Marker | Events | % Gated | Mean  |
|--------|--------|---------|-------|
| All    | 10000  | 100.00  | 64.62 |
| M1     | 8695   | 86.95   | 72.09 |

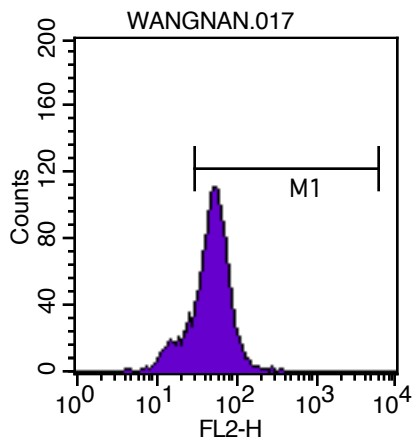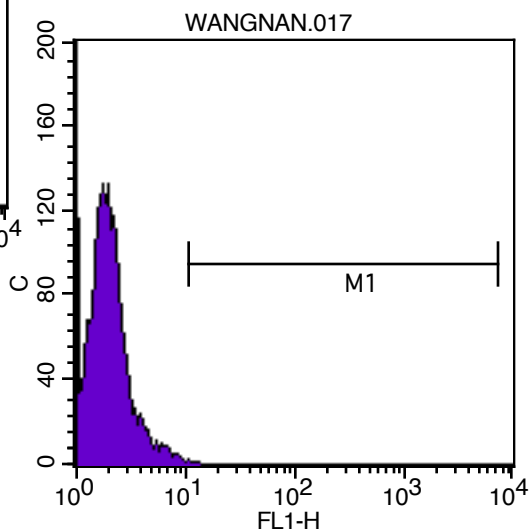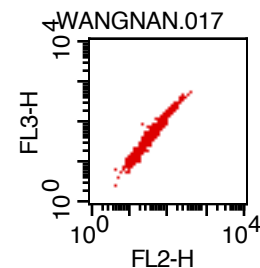

Supplement: Supplementary file 1 [file DataSheet1.ZIP › ROS/flow cytometry data/Controul/5.pdf]

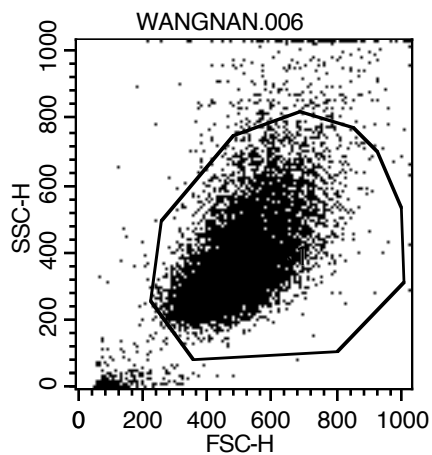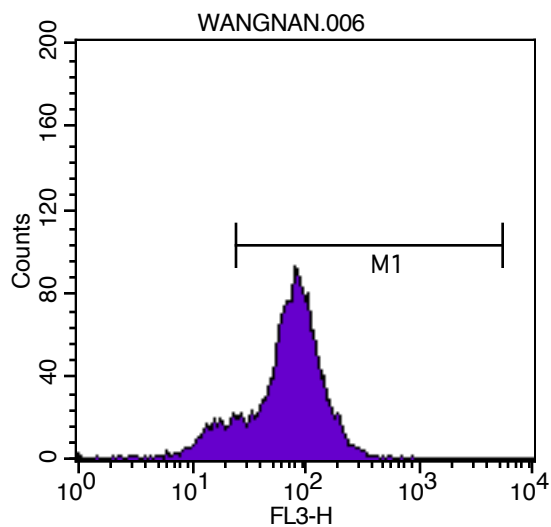

Gate: G1  
Total Events: 10722  
Gated Events: 1000  
X Parameter: FL3-H

| Marker | Events | % Gated | Mean  |
|--------|--------|---------|-------|
| All    | 10000  | 100.00  | 79.45 |
| M1     | 8941   | 89.41   | 86.94 |

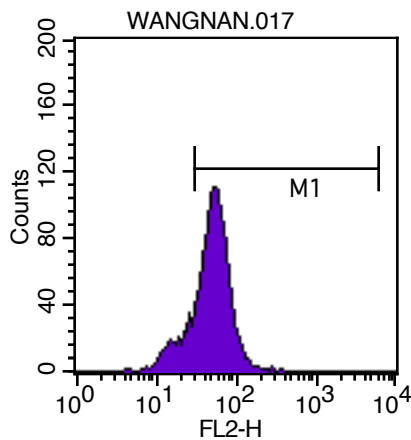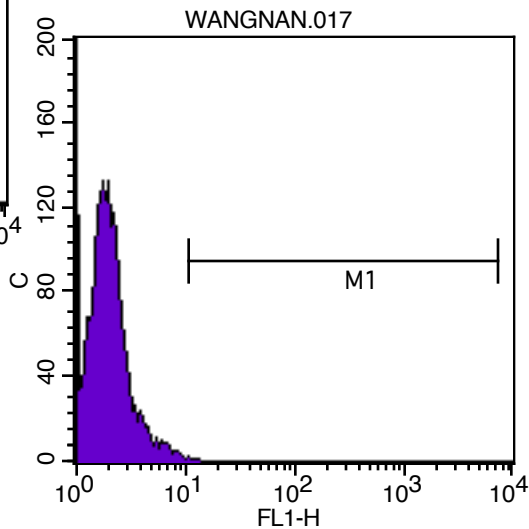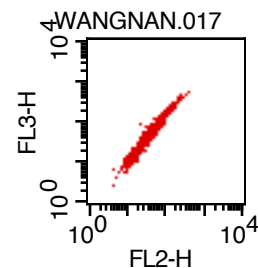

Supplement: Supplementary file 1 [file DataSheet1.ZIP › ROS/flow cytometry data/LPS/6.pdf]

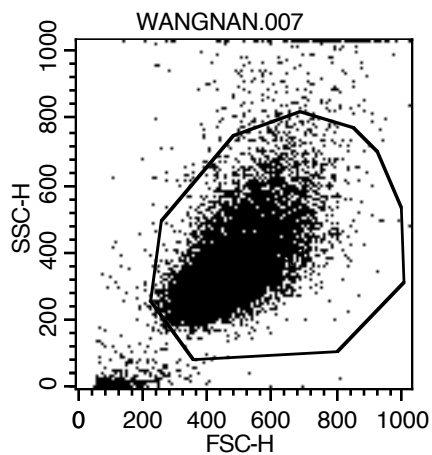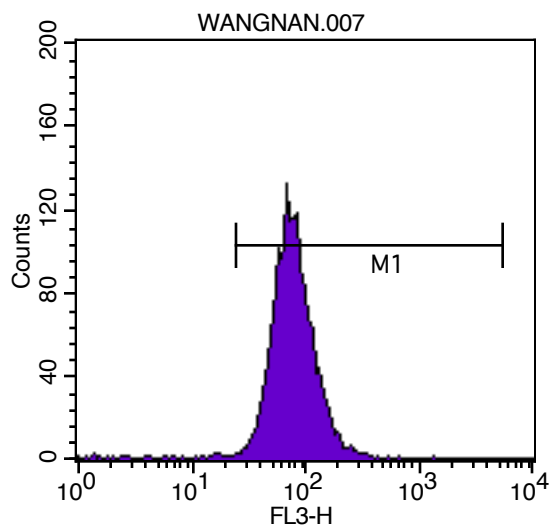

Gate: G1  
Total Events: 10845

Gated Events: 1000  
X Parameter: FL3-H

| Marker | Events | % Gated | Mean  |
|--------|--------|---------|-------|
| All    | 10000  | 100.00  | 80.54 |
| M1     | 9946   | 99.46   | 80.92 |

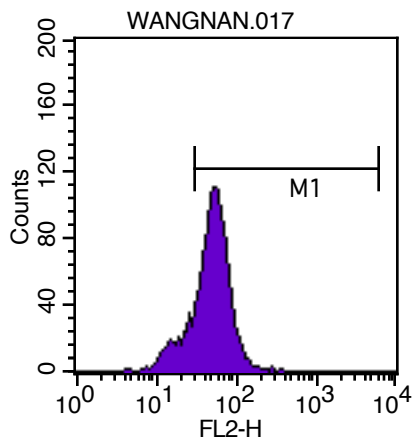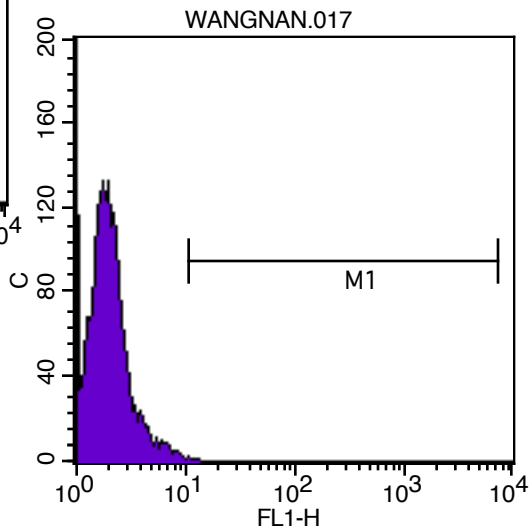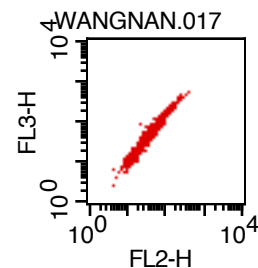

Supplement: Supplementary file 1 [file DataSheet1.ZIP › ROS/flow cytometry data/LPS/7.pdf]

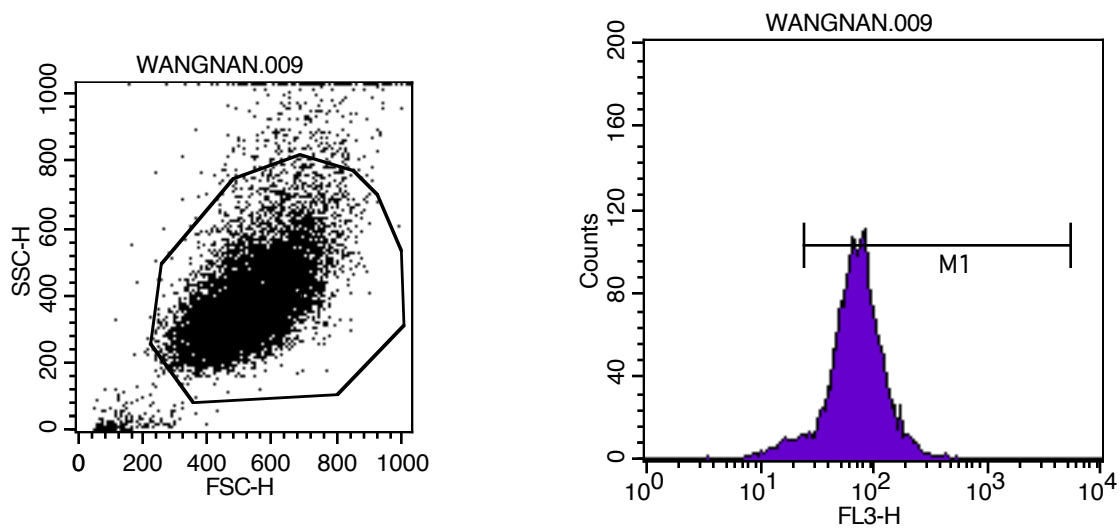

Gate: G1  
Total Events: 10617

Gated Events: 1000  
X Parameter: FL3-H

| Marker | Events | % Gated | Mean  |
|--------|--------|---------|-------|
| All    | 10000  | 100.00  | 75.10 |
| M1     | 9574   | 95.74   | 77.66 |

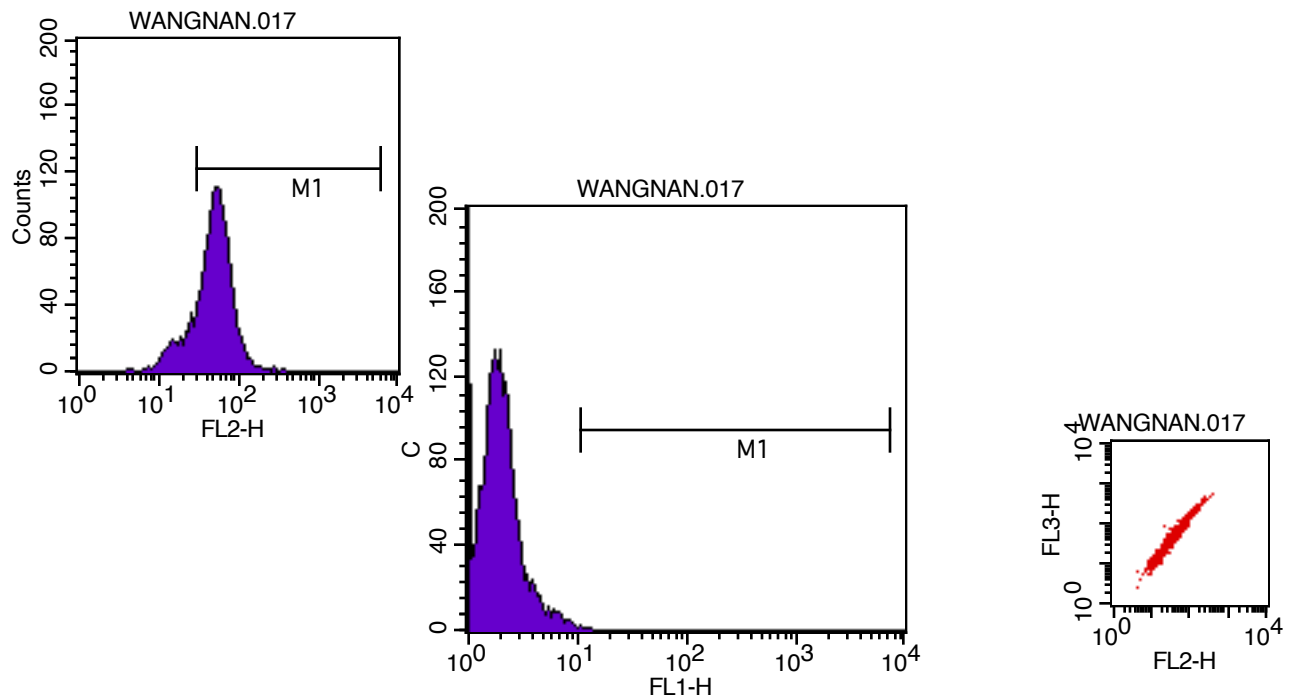

Supplement: Supplementary file 1 [file DataSheet1.ZIP › ROS/flow cytometry data/LPS/9.pdf]

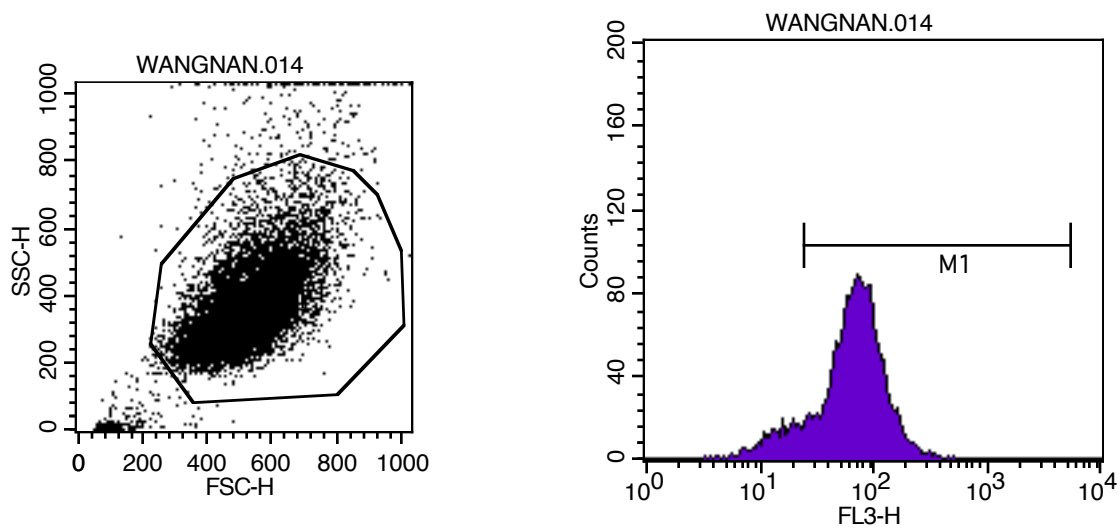

Gate: G1  
Total Events: 10524

Gated Events: 1000  
X Parameter: FL3-H

| Marker | Events | % Gated | Mean  |
|--------|--------|---------|-------|
| All    | 10000  | 100.00  | 71.53 |
| M1     | 9000   | 90.00   | 77.74 |

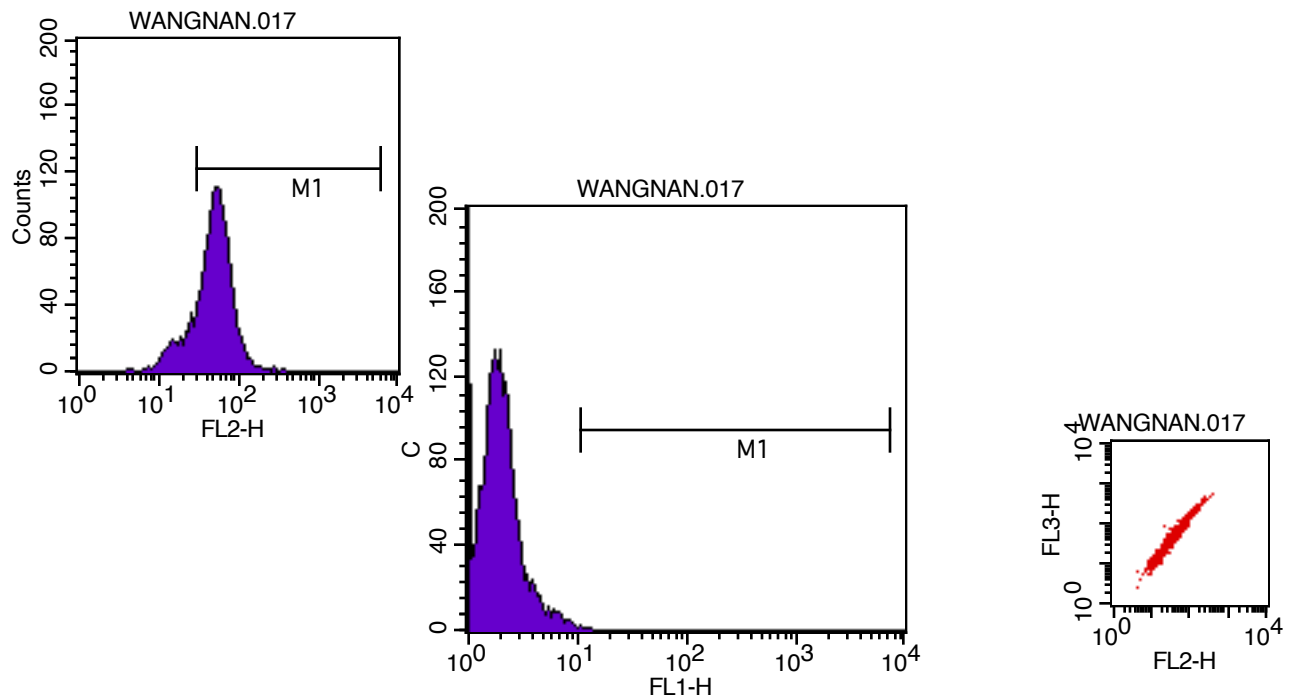

Supplement: Supplementary file 1 [file DataSheet1.ZIP › ROS/flow cytometry data/LPS+MNK/14.pdf]

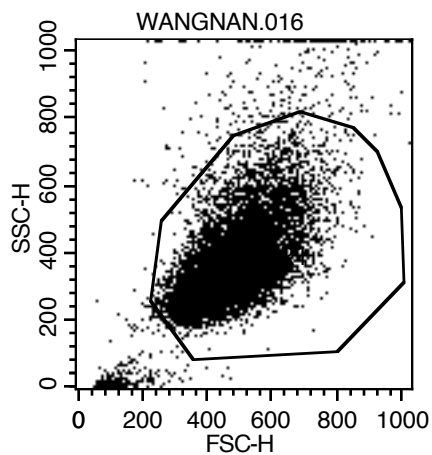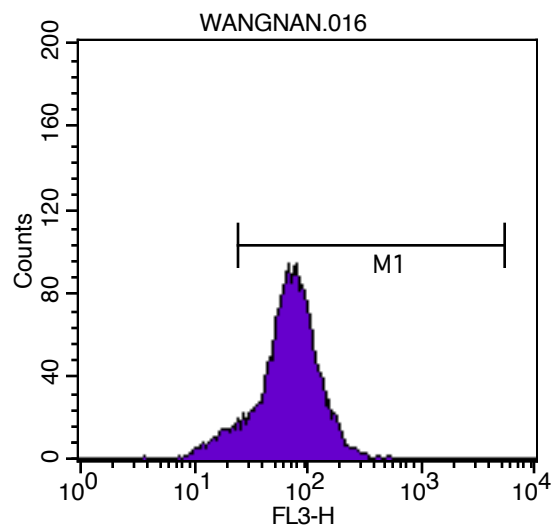

Gate: G1  
Total Events: 10649

Gated Events: 1000  
X Parameter: FL3-H

| Marker | Events | % Gated | Mean  |
|--------|--------|---------|-------|
| All    | 10000  | 100.00  | 74.74 |
| M1     | 9347   | 93.47   | 78.74 |

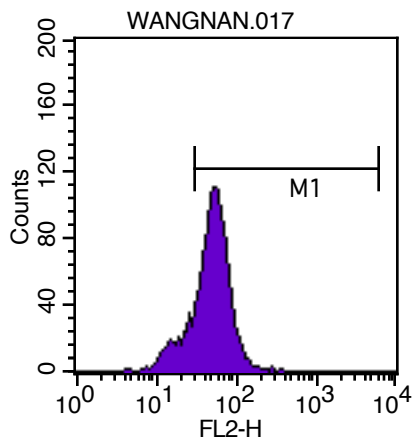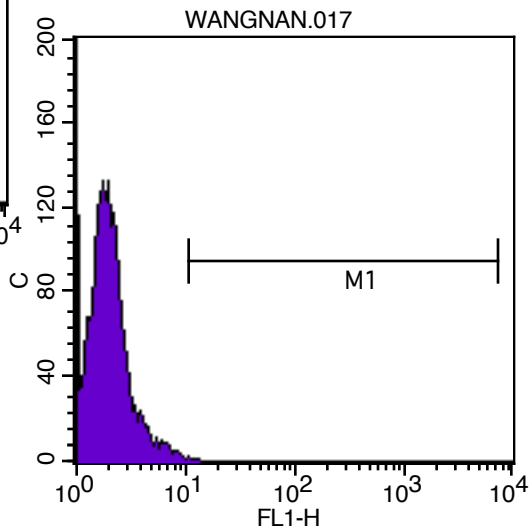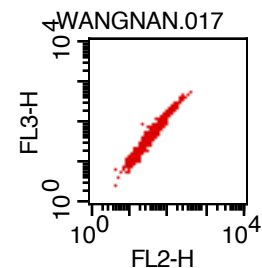

Supplement: Supplementary file 1 [file DataSheet1.ZIP › ROS/flow cytometry data/LPS+MNK/16.pdf]

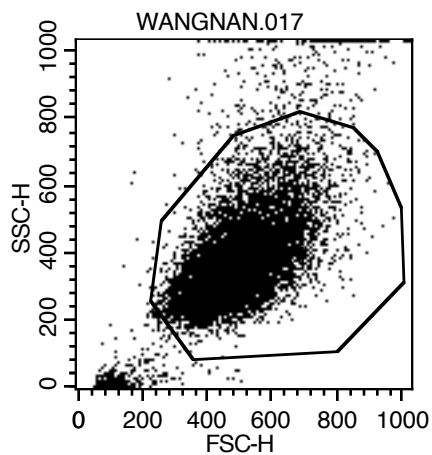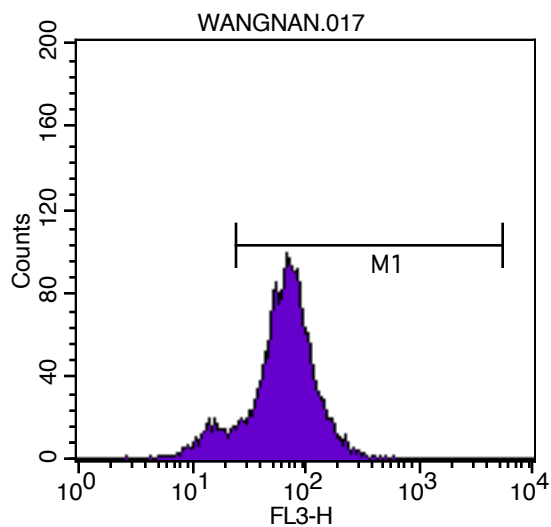

Gate: G1  
Total Events: 10772

Gated Events: 1000  
X Parameter: FL3-H

| Marker | Events | % Gated | Mean  |
|--------|--------|---------|-------|
| All    | 10000  | 100.00  | 70.21 |
| M1     | 9055   | 90.55   | 75.89 |

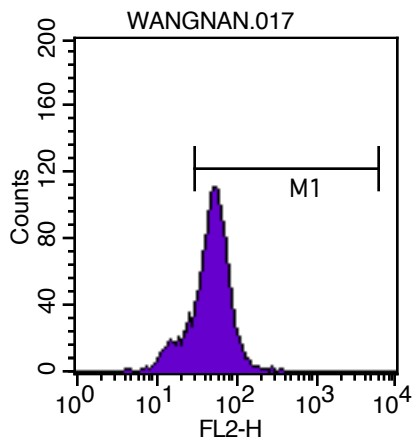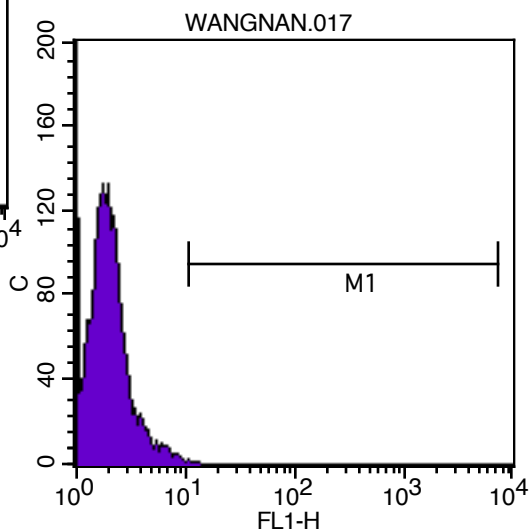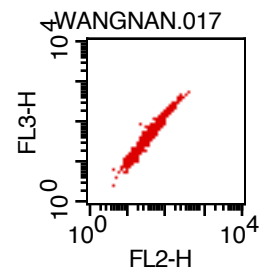

Supplement: Supplementary file 1 [file DataSheet1.ZIP › ROS/flow cytometry data/LPS+MNK/17.pdf]

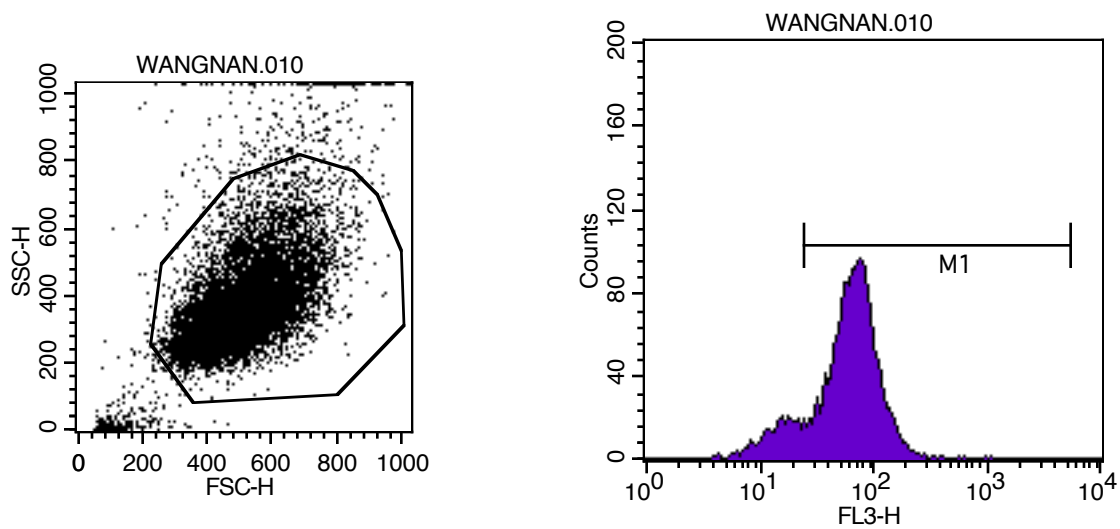

Gate: G1  
Total Events: 10714

Gated Events: 1000  
X Parameter: FL3-H

| Marker | Events | % Gated | Mean  |
|--------|--------|---------|-------|
| All    | 10000  | 100.00  | 64.73 |
| M1     | 8767   | 87.67   | 71.66 |

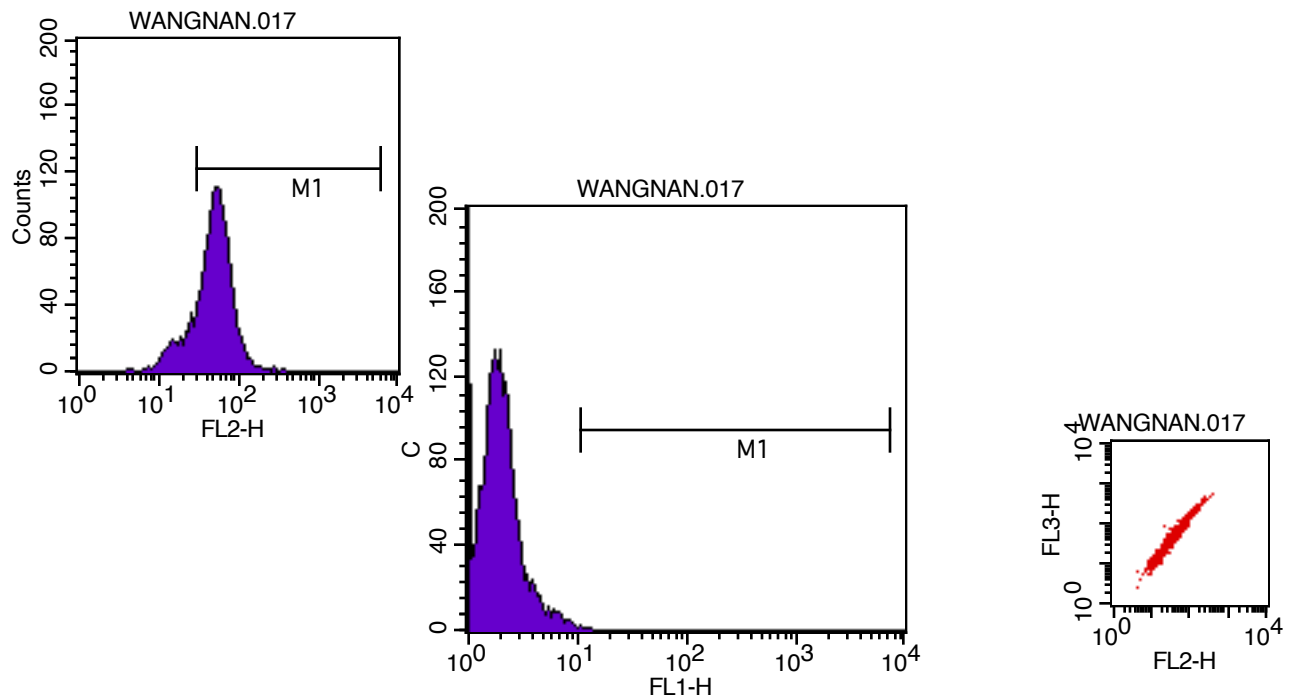

Supplement: Supplementary file 1 [file DataSheet1.ZIP › ROS/flow cytometry data/MNK/10.pdf]

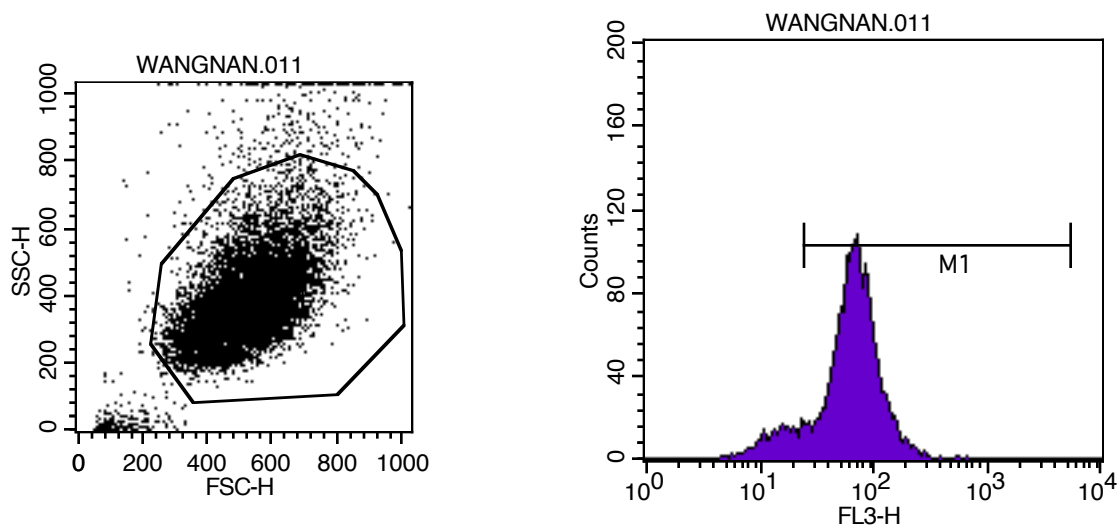

Gate: G1  
 Total Events: 10590  
 Gated Events: 1000  
 X Parameter: FL3-H

| Marker | Events | % Gated | Mean  |
|--------|--------|---------|-------|
| All    | 10000  | 100.00  | 67.22 |
| M1     | 9043   | 90.43   | 72.68 |

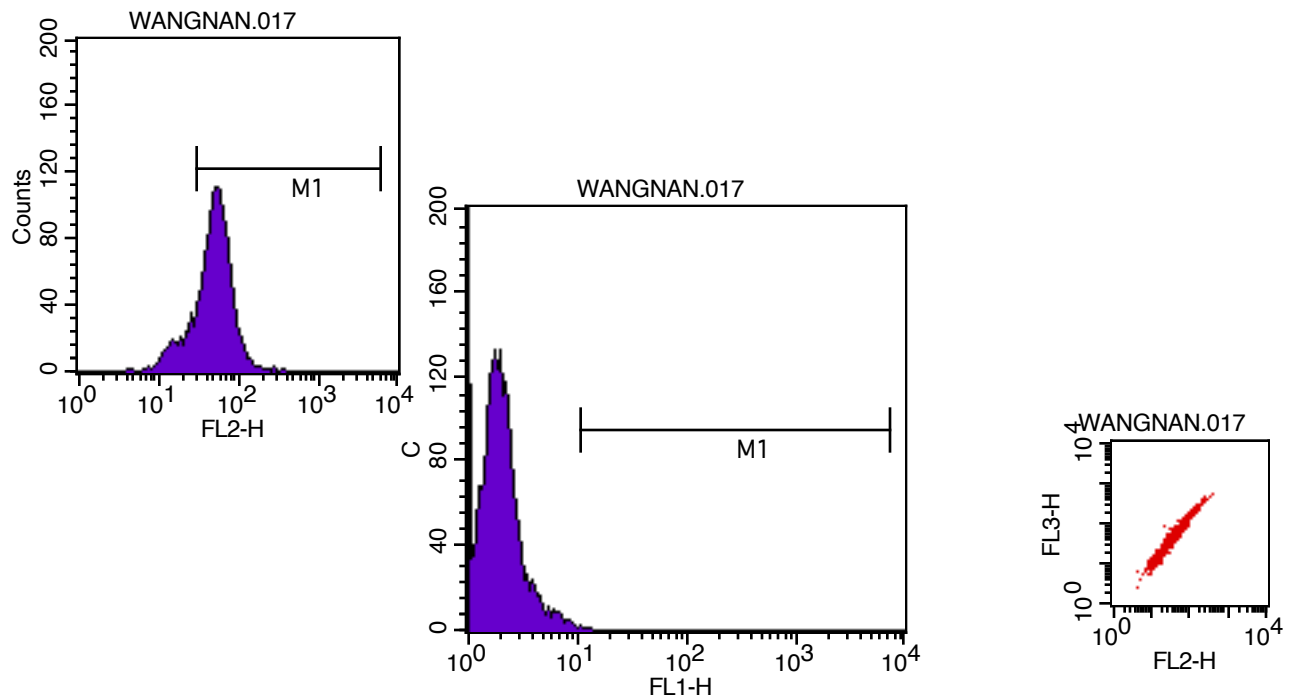

Supplement: Supplementary file 1 [file DataSheet1.ZIP › ROS/flow cytometry data/MNK/11.pdf]

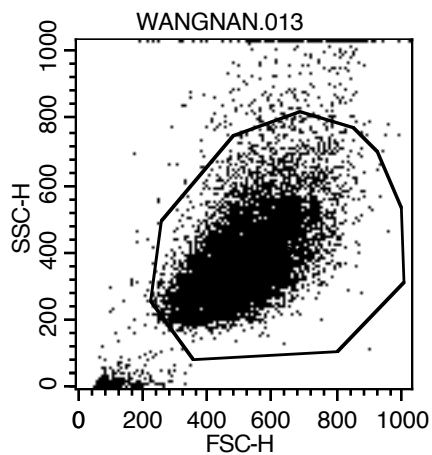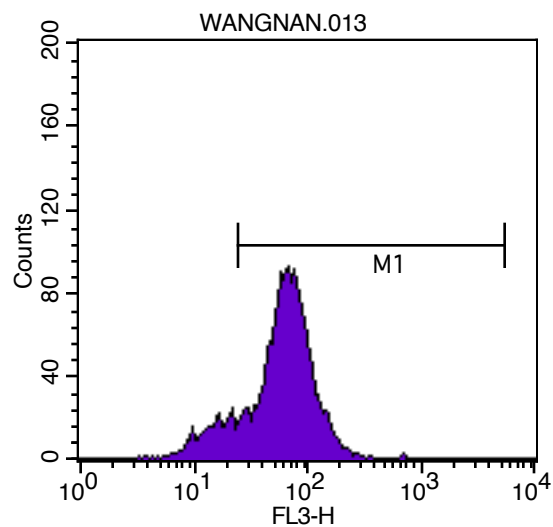

Gate: G1  
Total Events: 10680

Gated Events: 1000  
X Parameter: FL3-H

| Marker | Events | % Gated | Mean  |
|--------|--------|---------|-------|
| All    | 10000  | 100.00  | 64.58 |
| M1     | 8685   | 86.85   | 72.04 |

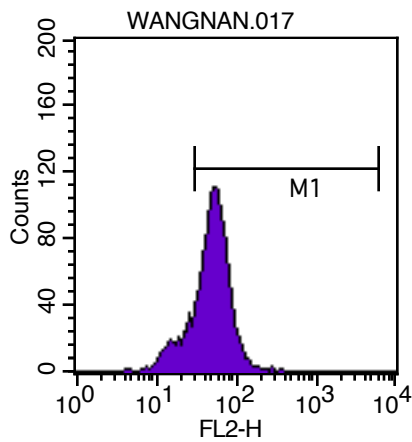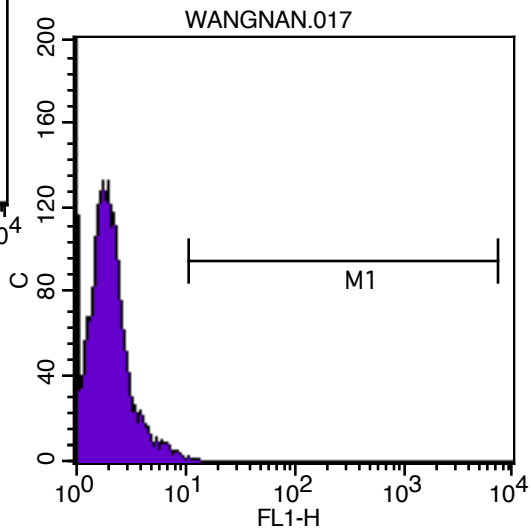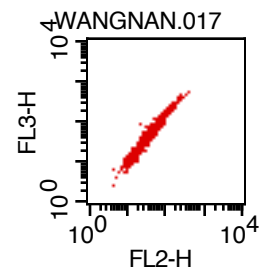

Supplement: Supplementary file 1 [file DataSheet1.ZIP › ROS/flow cytometry data/MNK/13.pdf]

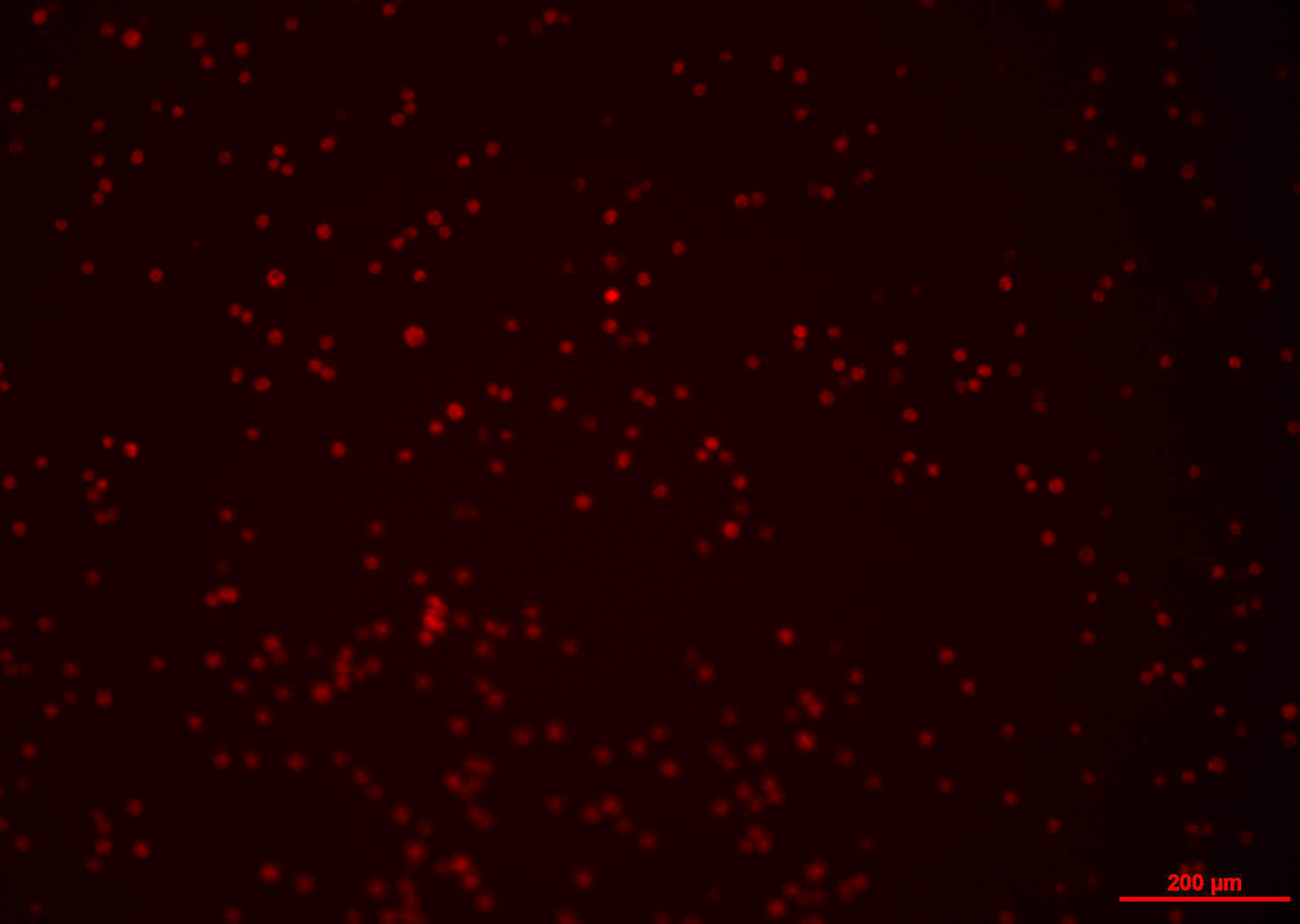

Supplement: Supplementary file 1 [file DataSheet1.ZIP › ROS/microscopy images/C.jpg]

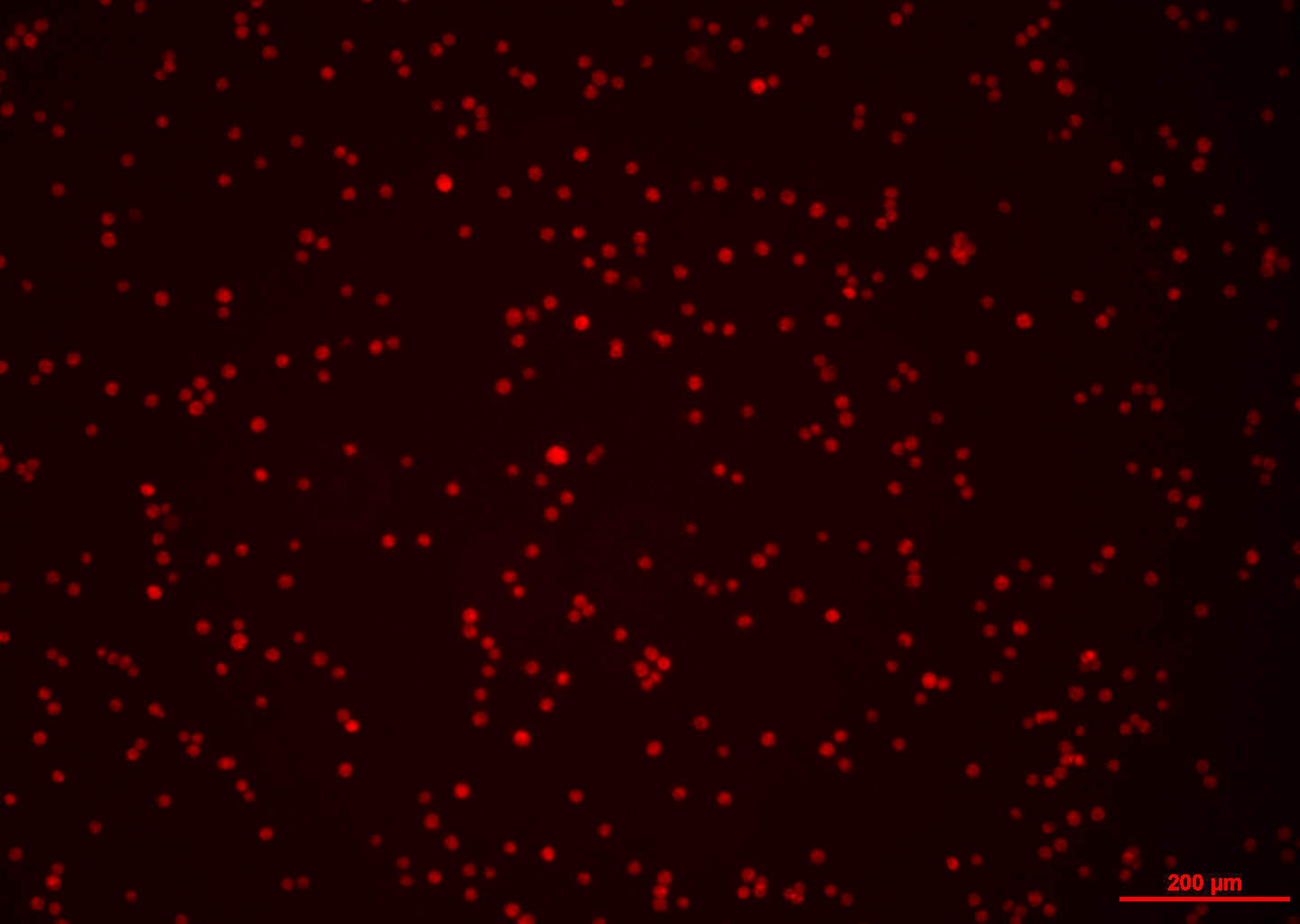

Supplement: Supplementary file 1 [file DataSheet1.ZIP › ROS/microscopy images/L.jpg]

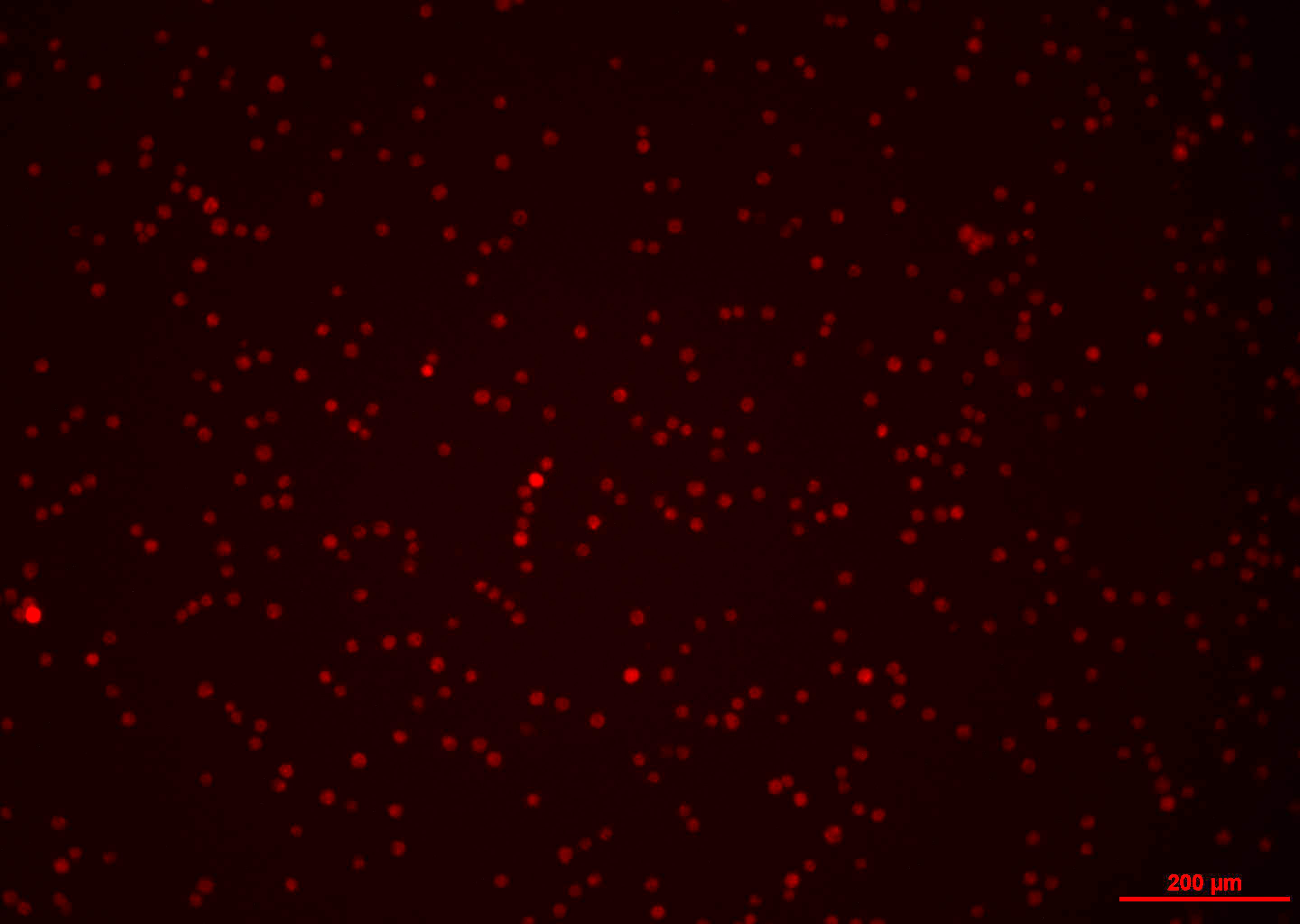

Supplement: Supplementary file 1 [file DataSheet1.ZIP › ROS/microscopy images/M+L.jpg]

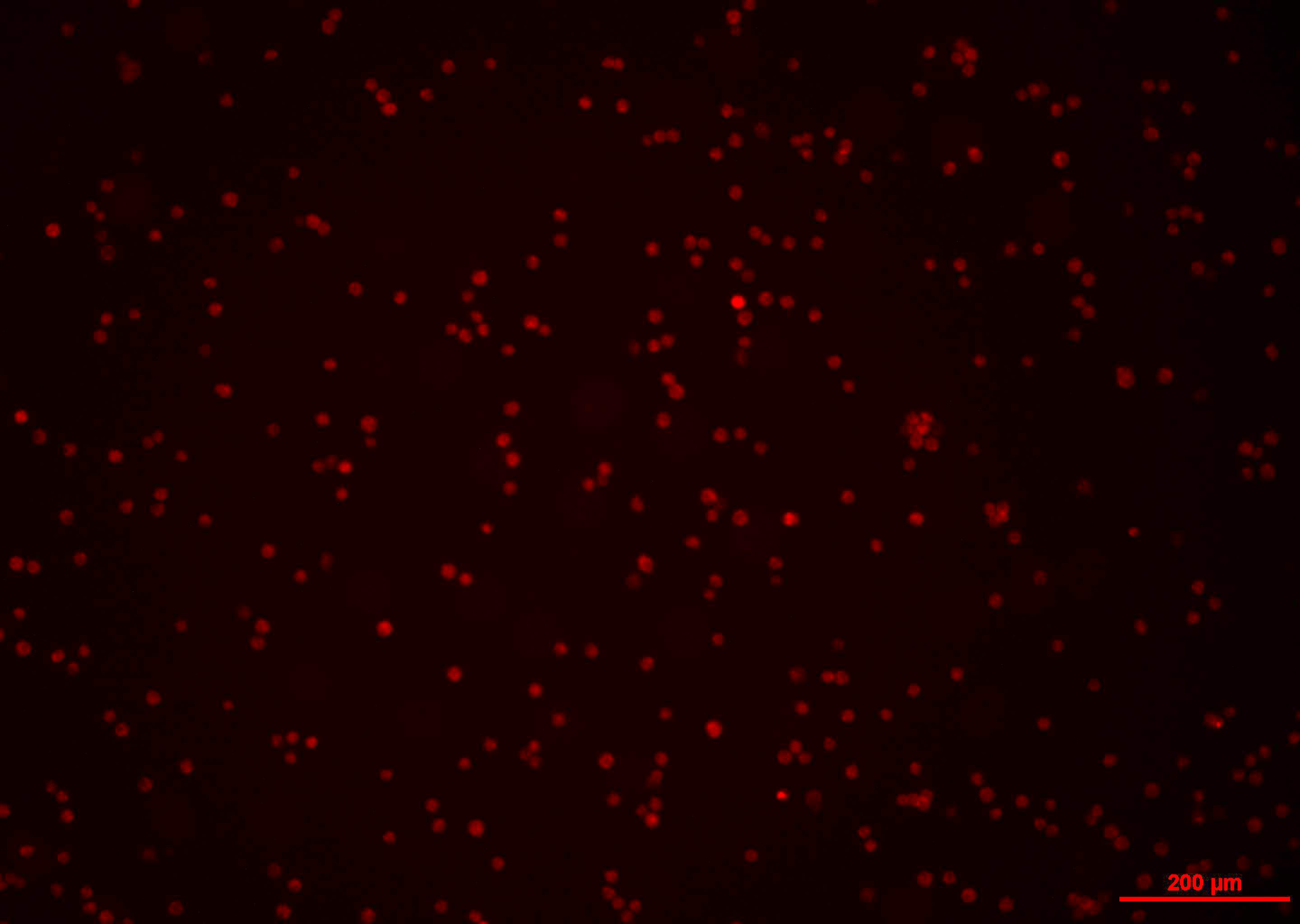

Supplement: Supplementary file 1 [file DataSheet1.ZIP › ROS/microscopy images/M.jpg]

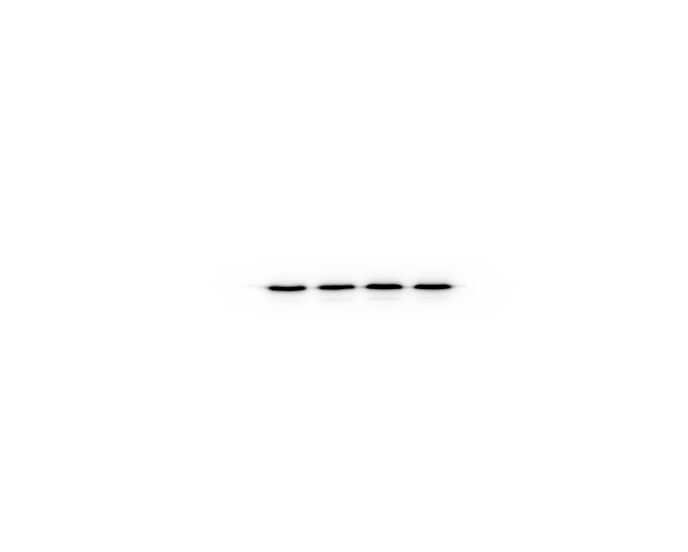

Supplement: Supplementary file 1 [file DataSheet1.ZIP › WB/Figure 5/1 GAPDH.tif]

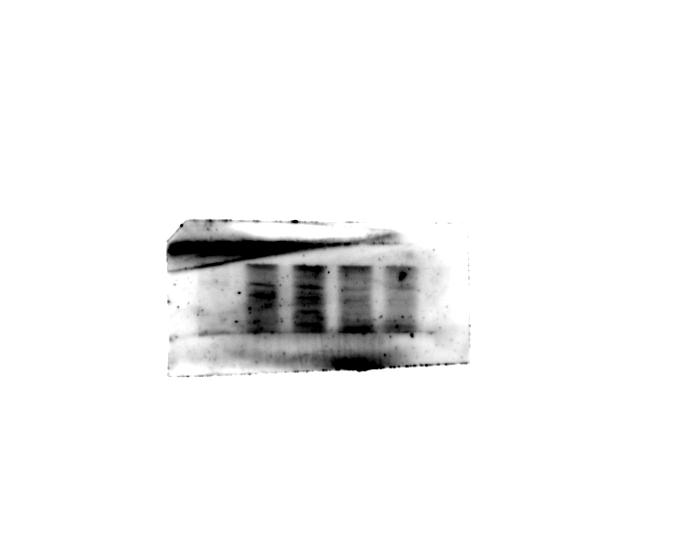

Supplement: Supplementary file 1 [file DataSheet1.ZIP › WB/Figure 5/1 IL6 .tif]

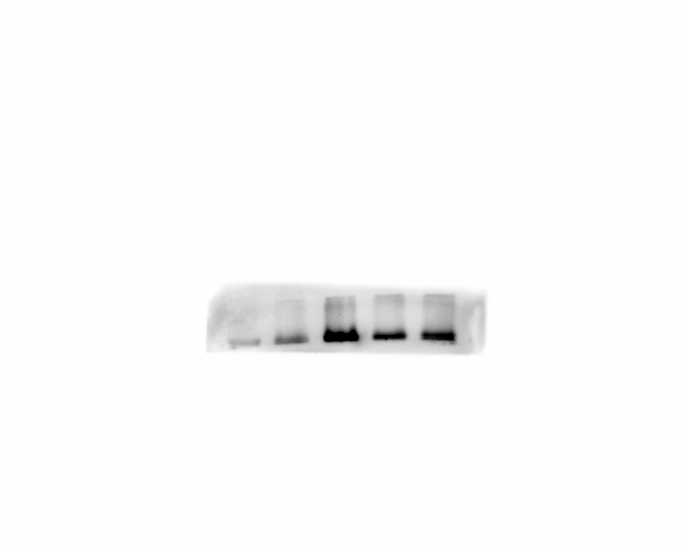

Supplement: Supplementary file 1 [file DataSheet1.ZIP › WB/Figure 5/1 STAT3.tif]

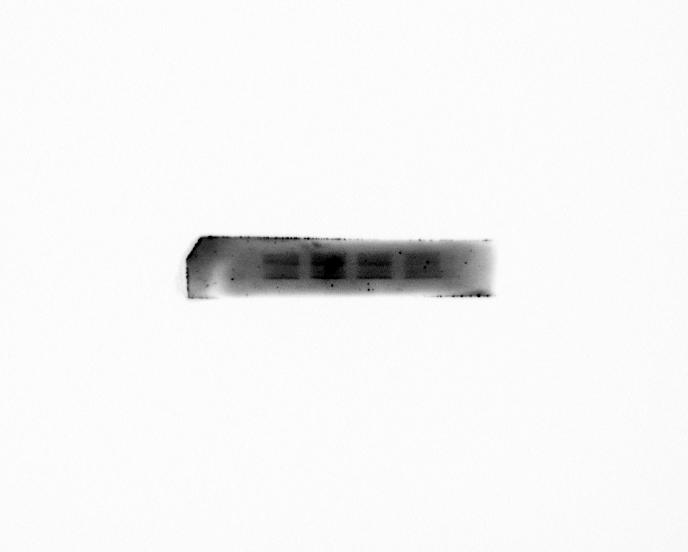

Supplement: Supplementary file 1 [file DataSheet1.ZIP › WB/Figure 5/1 TGFB .tif]

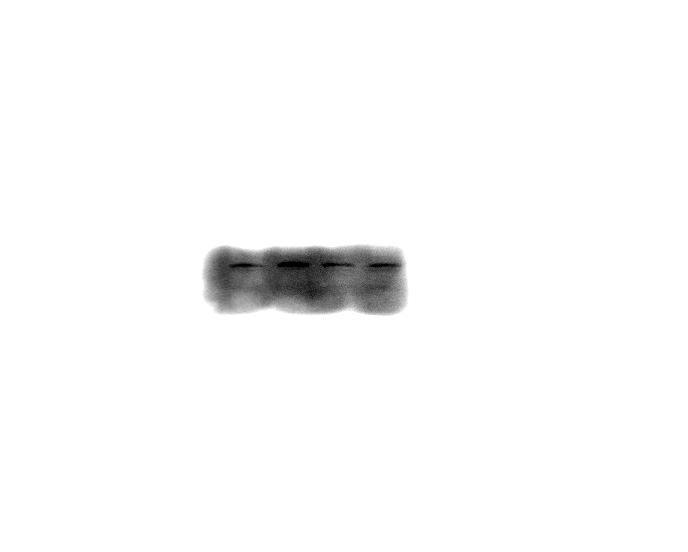

Supplement: Supplementary file 1 [file DataSheet1.ZIP › WB/Figure 5/1 TNFa.tif]

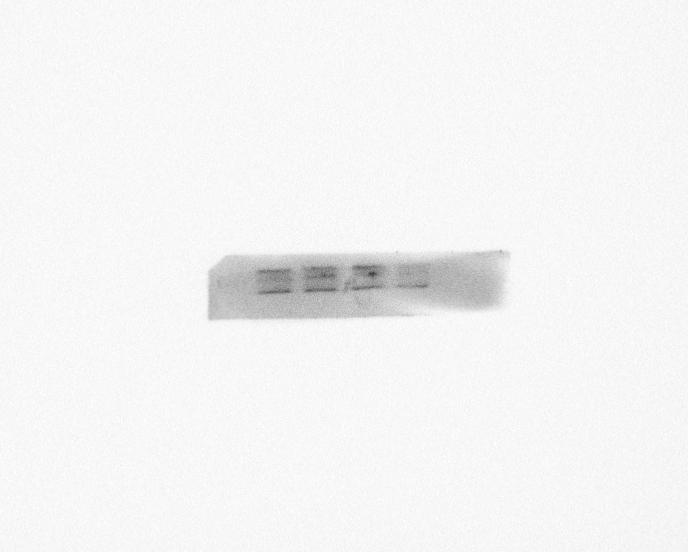

Supplement: Supplementary file 1 [file DataSheet1.ZIP › WB/Figure 5/1 TNFR1.tif]

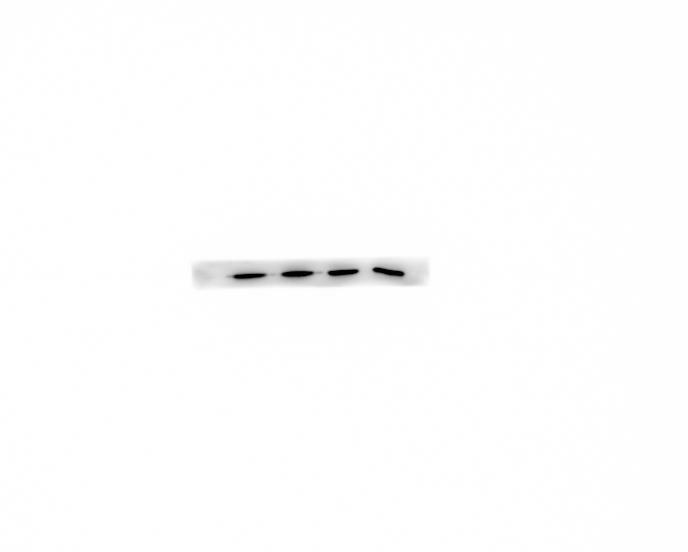

Supplement: Supplementary file 1 [file DataSheet1.ZIP › WB/Figure 6/2 GAPDH.jpg]

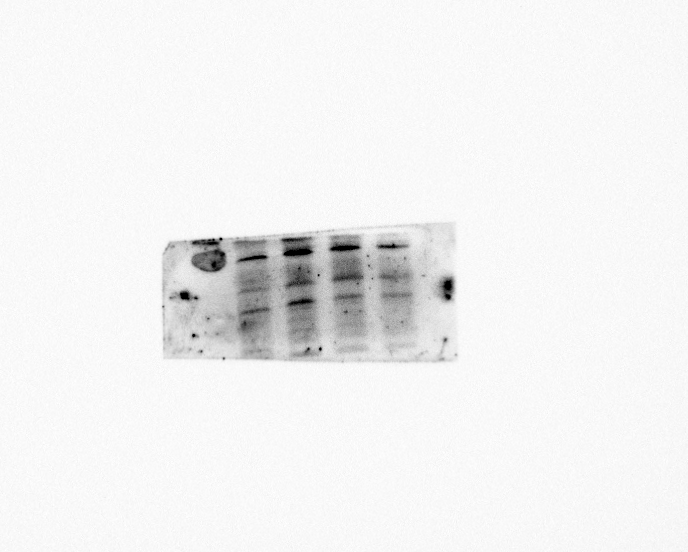

Supplement: Supplementary file 1 [file DataSheet1.ZIP › WB/Figure 6/2 MYD88.tif]

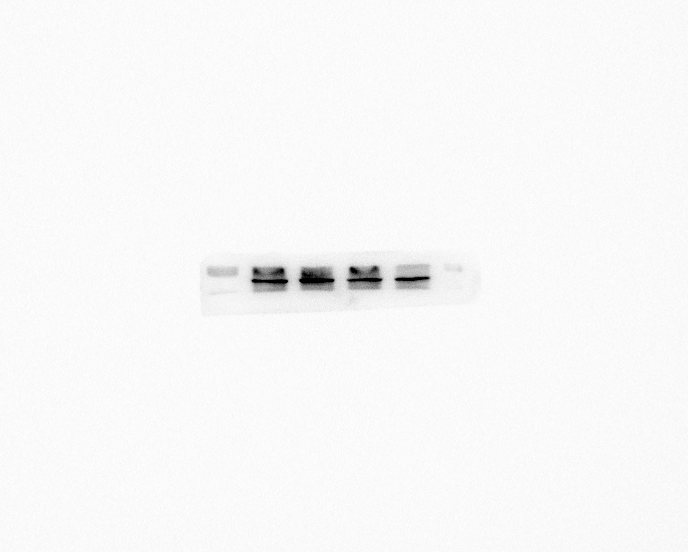

Supplement: Supplementary file 1 [file DataSheet1.ZIP › WB/Figure 6/2 p-p65.tif]

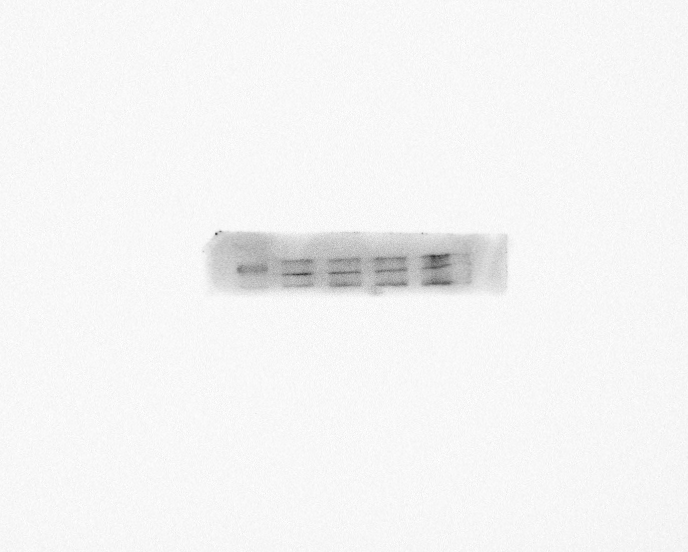

Supplement: Supplementary file 1 [file DataSheet1.ZIP › WB/Figure 6/2 p65.tif]

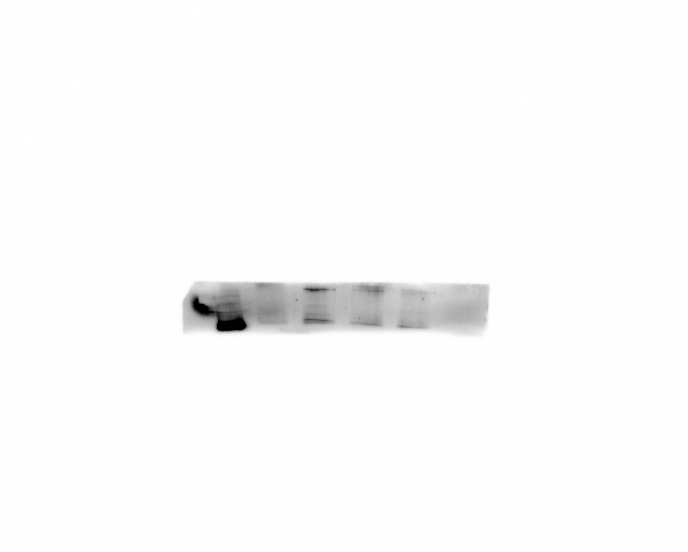

Supplement: Supplementary file 1 [file DataSheet1.ZIP › WB/Figure 6/2 TLR4.tif]

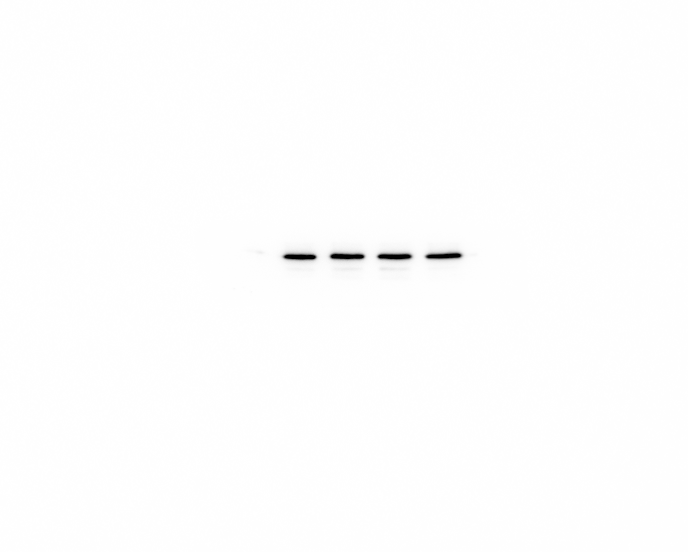

Supplement: Supplementary file 1 [file DataSheet1.ZIP › WB/Figure 7/1 GAPDH.tif]

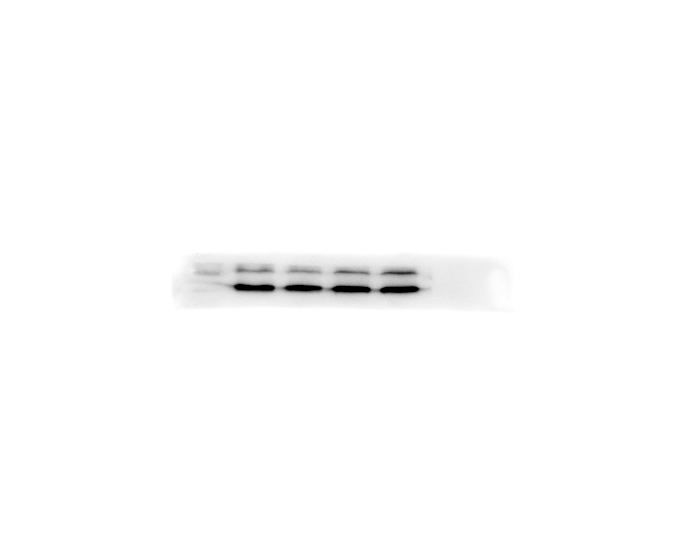

Supplement: Supplementary file 1 [file DataSheet1.ZIP › WB/Figure 7/1 P-AKT.tif]

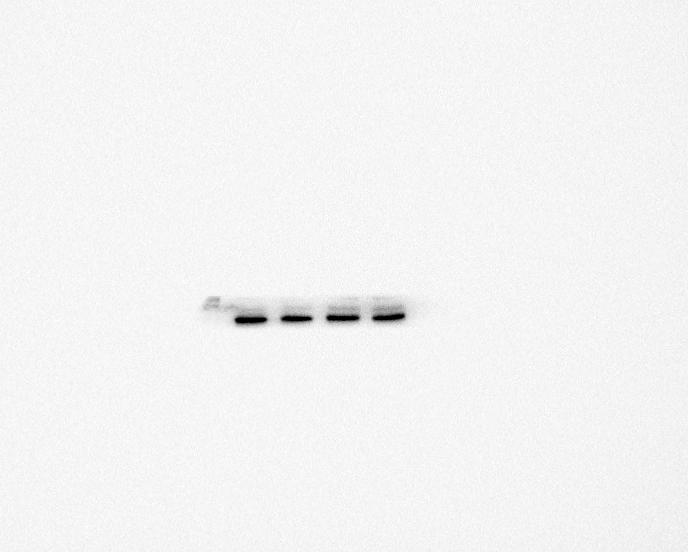

Supplement: Supplementary file 1 [file DataSheet1.ZIP › WB/Figure 7/2 ERK .tif]

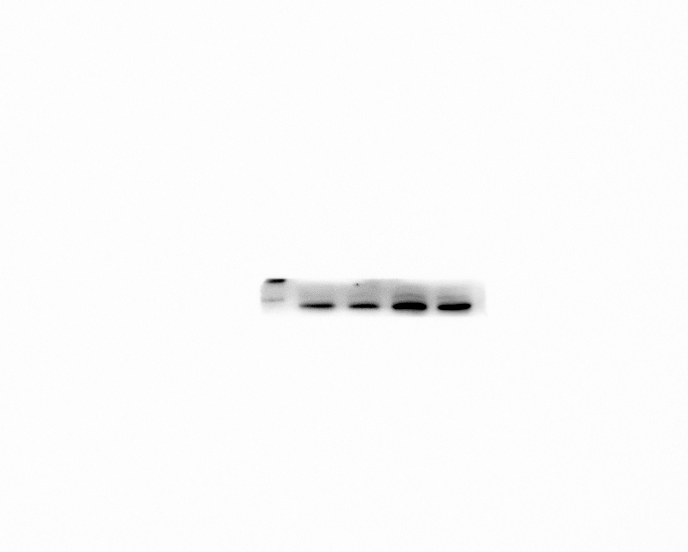

Supplement: Supplementary file 1 [file DataSheet1.ZIP › WB/Figure 7/2 P-ERK.tif]

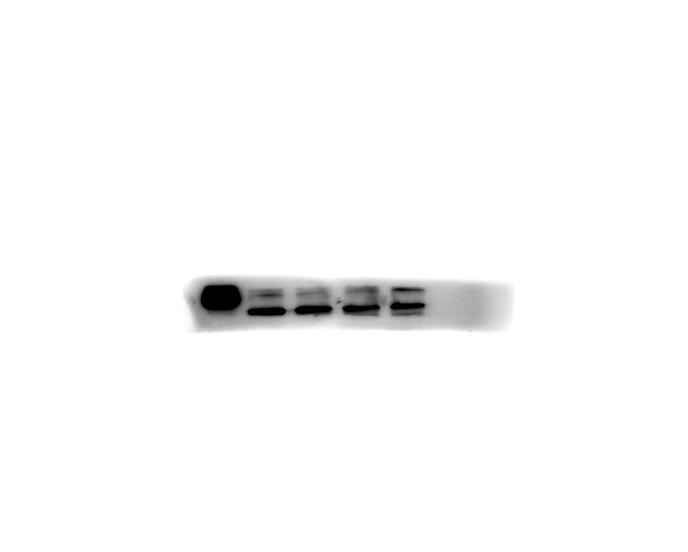

Supplement: Supplementary file 1 [file DataSheet1.ZIP › WB/Figure 7/3 AKT .tif]

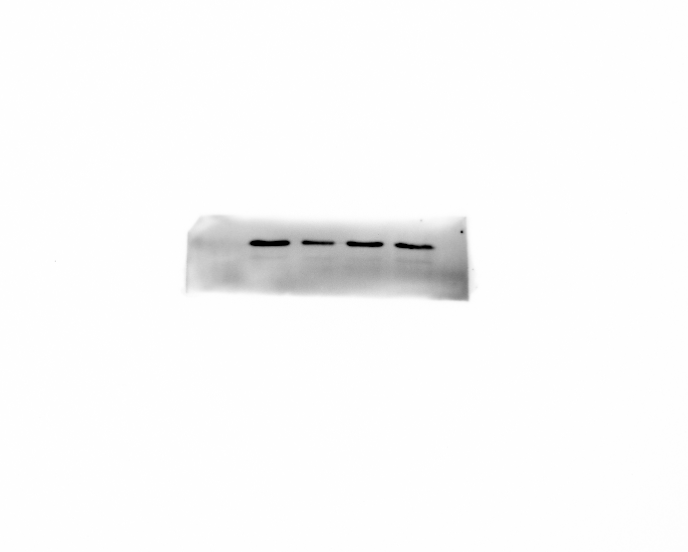

Supplement: Supplementary file 1 [file DataSheet1.ZIP › WB/Figure 7/3 Nrf2 .jpg]

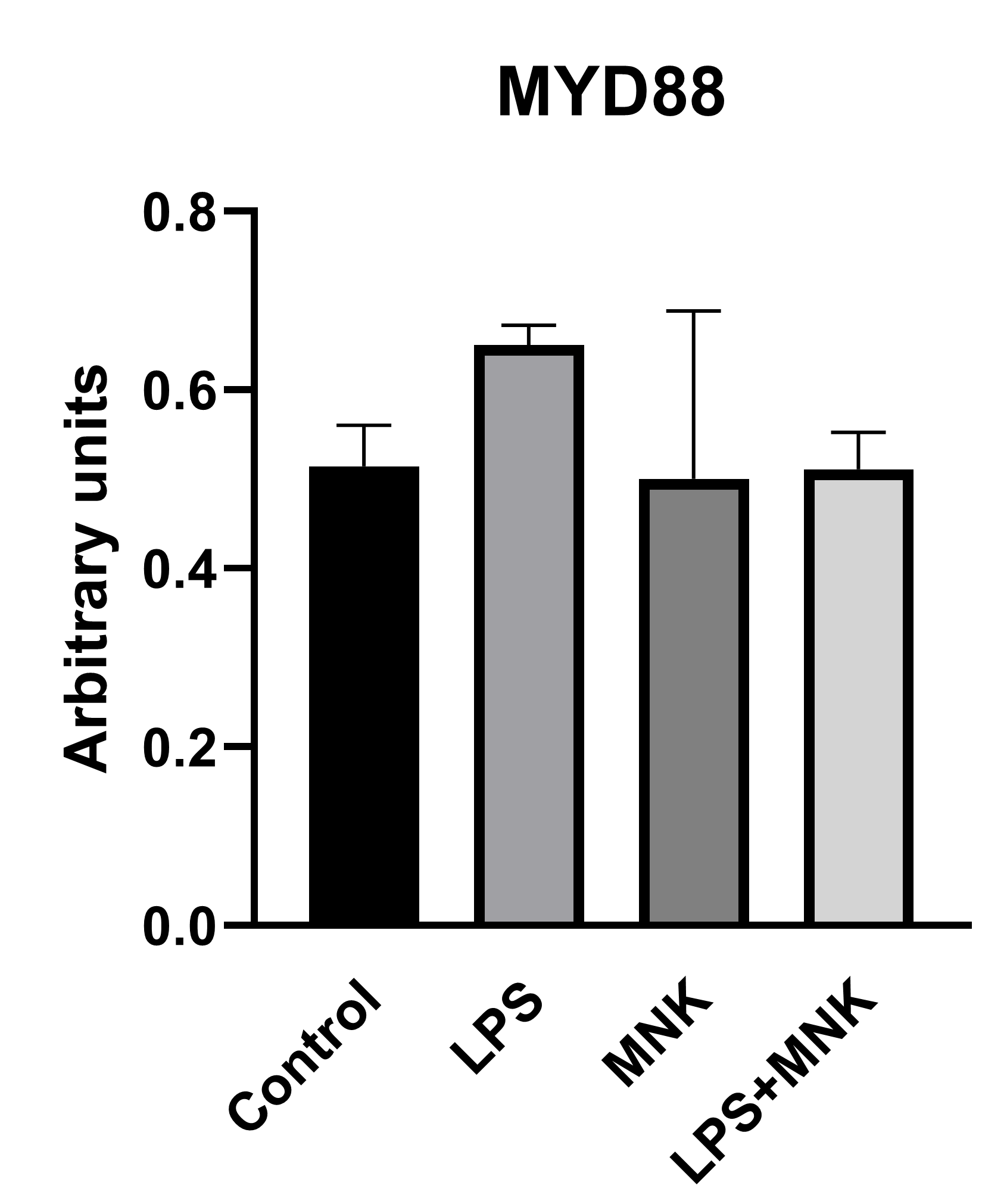

Supplement: Supplementary file 2 [file Image1.TIF]
