# Supplementary material for: 2-Methyl Nonyl Ketone From Houttuynia Cordata Thunb Alleviates LPS-Induced Inflammatory Response and Oxidative Stress in Bovine Mammary Epithelial Cells
Source: Front Chem. 2022 Jan 31;9:793475. doi: 10.3389/fchem.2021.793475 (PMC8842123; doi:10.3389/fchem.2021.793475)
Supplement: Supplementary file 3 [file Table2.docx]

**Table 2.** English abbreviation

| **English abbreviation** | **English full name** |
| --- | --- |
| *H.cordata* | *Houttuynia Cordata Thunb* |
| MNK | 2-methyl nonyl ketone |
| LPS | lipopolysaccharide |
| MAC-T | bovine mammary epithelial cell line |
| ROS | reactive oxygen species |
| IL | interleukin |
| TNF-α | tumor necrosis factor-α |
| STAT3 | signal transducer and activator of transcription |
| TLR4 | Toll-like receptor 4 |
| NF-κB | nuclear factor-κB |
| HO-1 | Heme Oxygenase-1 |
| Nrf2 | Nuclear factor erythroid 2-related factor 2 |
| ERK | extracellular signal-regulated kinase |
| qPCR | quantitative real-time PCR |
| SD | standard deviation |
| TNFR1 | TNF-receptor-associated complex I |
| MYD88 | myeloid differentiation factor 88 |
